# General Index to the British and Foreign Medical Review

**Published:** 1848

**Authors:** 


Cæsarean section, cases of . . xx 534

causes of death in . . xiv 202

dates of death after . xiv 202

Dr. Kayser on . . xiv 199

four times performed . iv 521

history of . . . xiv 200

mortality from . . xiv 200

report on . . . xvii 539

remarks on . . . ix 39

successful cases of . ii 568, x 578, xxi 274, xxiii 284

success of . . . xii 489

suggestion on . . iv 521

Caffein not poisonous . . xiv 308

possible use of to the brain . xiv 308

the alkaloid of tea and coffee xiv 308

Caffeine, identity of with theine . xiv 517

Caffe, M., on ophthalmia . . ix 557

Caïnca, a new remedy, account of v 562

medicinal powers of . . v 562

Cairo and Alexandria, improvement of xxiv 244

hospital and medical school of vi 592

medical institutions of . . vi 592

plague in 1834-5 . . . i 248

in a factory not far from xxiv 249

Calamus scriptorius, opening at the xiv 464

Calcareous bodies in the ear . . viii 81

state of placenta, case of . v 115

tumours, analysis of . . ii 164

Calcium as an element of food . xvii 4

Calculating machine, Babbage's . xix 161

Calculation, observations on . xii 4

scientific, importance of . xii 20

Calculi, alternations of deposits in . ix 368

biliary, composition of . . xvii 447

jaundice, &c. . . xx 244

varieties of . . . xvii 446

breaking up, fatal case of . xii 421

carbonate of lime . . . ix 366

chemical constitution of 277 . iii 255

relations of . . . ix 360

solvents of . . . ix 368

classification of . . . ix 367

colour of . . . . ix 361

crushing, Weiss's inventions for xii 410, 415

deposition and increase of . ix 368

Egyptian operation for . . xii 400

form of . . . . . ix 360

history of . . . . ix 360

in children, frequency of . xiv 165

in peculiar localities . . xiv 165

in St. George's Hospital . . xviii 366

intestinal, L'Heritier on . . xvii 446

lithic acid . . . . ix 363

magnesian . . . . ix 365

mode of examining . . ix 362, 367

nuclei of . . . . ix 362

abstract view of . . xvi 313

odour of . . . . ix 362

oxalate of lime . . . ix 366

production of . . . xiv 308

phosphate and subphos. of lime ix 365

removal of by catheters . . xii 402

through urethra . . xii 400

Calculi, removed by forceps through urethra . . . . xii 403

through dilated urethra xii 400

through female urethra xii 402

size and weight of . . . ix 361

spontaneous breaking down of xiv 166

structure of . . . . ix 362

surface of . . . . ix 361

urinary, causes of . . ix 368

frequent in India . . xii 425

L'Heritier on . . . xvii 447

on a piece of straw . xii 332

use of alkalies in . . . ix 368

vesical, Dr. Scharling on . xv 537

xanthic oxide . . . ix 364

Calculous concretions . . xvii 446

deposits in kidneys . xv 472, 480

diseases, Dr. Civiale on . . ii 276

Dr. Prout on . . . xi 330

in Mauritius . . xvii 288

in seamen . . . vii 141

in tropical climates . xx 215

new remedy in . . xiii 264

statistics of . . . ii 276

Calculus, a remarkable, in Guy's museum iv 376

benzoic acid in . . . xii 398

Berzelius on solution of . . vii 167

bronchial, case of . . . xviii 365

case of a large biliary . . xviii 366

caution to physicians on . vii 166

cures of, by Vichy waters . xii 396

effects of alkalies on . . vii 167

simple diluents on . . vii 166

Vichy waters on . . vii 166

empirical remedies for . . xii 391

gravel and gout, Dr. Jones on xv 500

Gruithuisen's paper on . . xii 405

in submaxillary duct, case of . ix 248

large, in a female child . . xiv 255

lachrymal, analysis of . . xv 239

lithic acid, on alkalies in . xi 354

treatment in . . . xi 353

medicines by the mouth for . xii 390

notice on etiology of . . viii 150

pulmonary . . . viii 279

scrotal, Prof. Chelius on . ii 260

solution of . . . vii 166

solution of a large . . vii 167

by injections . . vii 167

by nitric acid . . vii 168

by Sir B. Brodie . . vii 168

statistics of, in Austria . . iii 254

sulphur in cystic oxide . . xii 458

treatises on . . . . xii 388

treatment of by injections . xii 398

urethral, large, case of . . x 264

urinary, on a remarkable . iv 375

use of alkalies in . . xii 390

bi-borate of soda in . xii 398

borax in . . . ix 369

Vichy waters in . . xii 393

Calculus of probabilities explained . xii 19

use of the . . . xii 18

Calcutta, epidemics of . . . xvi 207

Calcutta Med. Society, transactions of iii 329
mortality in, from smallpox . xx 350
native hospital, diseases in . viii 592
sanitary improvement of . xvi 206
CALDERHEAD, Mrs., fatal case of . v 178
CALDER, Mr., on squinting . xi 474, 492
CALDWELL, Dr., on malaria . . i 349
on mesmerism . . xix 428, 449
on physical education . . iii 472
Calendula plant in uterine disease . xxi 472
Callisthenic exercises of females . v 407
Callipers, Dr. Stokes's, for the chest iv 322
of Baudelocque . . . i 106
CALLISEN, Dr., his lexicon of medical authors . . . xvii 504
on artificial anus . . . xviii 462
Callus, absence of in the cranium . iii 77
absorption of, in typhus . xi 253
on the formation of . . xxiv 405
Calomel and black draught, abuse of v 390
and cotton in ophthalmia . xx 463
and opium in iritis . . ii 348
conversion of, by alkaline chlorides . . . . xviii 545
effect of common salt on . xviii 545
endermic use of . . . v 352
external use of, in ophthalmia v 251
given in tablespoonfuls . . xxii 235
in fever, neglect of in Paris . ii 63
in ophthalmia neonatorum . xiv 554
M. Valleix's strictures on . xviii 290
snuffed into the nostrils . xxiii 365
use and abuse of . . . xxii 188
Caloptrical exploration of the eye . x 8, 24
CALORI, Dr., on the structure of brain v 220
Caloric and electricity, on identity of xviii 518
Dr. Metcalfe on . . . xviii 513
experiment on, with ice . . xviii 515
generation of, in the system . xviii 520
in relation to vital actions . xviii 518
Mr. Exley's theory of . . xviii 514
material theory of . . xviii 515
not mere motion nor vibration xviii 514
Calorifiant matter in food, absence of xxiii 500
Calor mordax in fever electrical . viii 445
Calotype, Mr. F. Talbot's, French claim to . . . . xxiv 400
Cambric muslin obstructs percussion iv 293
Cambuslang, epidemic chorea at, 1742 xii 107
Camelidæ, blood-corpuscles of the . xvi 287
spermatozoa of the . . xvi 287
Camel, on smallpox in the . . x 281
Camels drunk, from dates and water xxiii 160
Camel's flesh, use of as food . . xvii 16
CAMERER, his celebrated experiments ii 551
Camomile flowers in ague . . xxi 212
Camp and typhoid fever, identity of xii 46
CAMPBELL, Dr. J. S., his cases of phthisis xiv 195
his literary carelessness . . xiv 196
on phthisis, his style . . xiv 196
on tuberculous phthisis . . xiv 181
summary of his theory . . xiv 190
CAMPBELL, Dr. W., his treatise on midwifery . . . xx 208
CAMPBELL, Dr. W., on extra-uterine gestation . . . . x 247
CAMPBELL, Mr. P., on ischuria renalis ix 573
CAMPER, facial angle of . . ix 209
his facial angle, remarks on . xviii 446
Camphine, a compound radical . xi 134
Camphor, a panacea . . . vii 549
fever from . . . . v 271
its effect on the heart . . i 264
no antidote to cantharides . ii 549
poisonous to insects . . iv 130
tympanites cured by . . ii 552
use of, in diseases of the heart iii 510
valuable properties of . . xvi 360
vinegar, an antidote against . i 244
Camp meetings in America . . iv 59
Canada, diseases of lungs in . . viii 222
Canaliculi and lacunæ of bone xxiv 389, 393-5
Canalis arteriosus, deviations of . xix 495
Canal of Wharton, case of calculus in ix 248
Cancer, a general disease . . xi 166
analogies of the species of . xi 158
anatomical appearances of . iii 65
case of, by Dr. Walshe . xxi 223
cause of intense pain in . iii 24
chemistry of . . . xxi 426
chimney-sweeper's . . xvii 87
colloid, anatomy of . . xi 161
characteristics of . . xi 160
varieties of . . . xxi 425
common, of the face . . vii 144
constitutional character of . xxiv 378
course and symptomatology of xxi 430
cure and curability of . . xi 165
of by compression . xi 166, xiv 13
deaths from in England . . ix 356
at different ages and sexes xi 165
decay of . . . . xxi 428
Dr. Carswell on . . . i 127
Dr. Hannover on . . . xxiv 373
Hannover's definition of . xxiv 374
Dr. Lebert on . . . xxii 32
Dr. Walshe on . . . xi 158
on the growth of . . xi 162
on the origin of . . xi 162
Dr. Walshe's definition of . xi 158
treatise on . xxi 421, xxii 293
duration and etiology of . . xxi 431
effect of compression on . xxi 220
encephaloid, characters of . xi 160
varieties of . . . xxi 424
endermic use of morphium in ii 517
extraordinary dictum on . xvi 272
gelatiniform, case of . . xx 100
general pathology of . . xxi 428
growth of . . . . xxi 427
hereditary, on operating in . xiv 14
important practical remarks on iii 65
Lisfranc on, criticised . . xiv 12
Lisfranc's use of hemlock in . xiv 15
medullary, probably scrofulous ii 20
mortality from, in ages and sexes xi 164
nature and origin of . . xxiv 377
progress of . . . xxi 219

Cancer, nature and structure of . x 235

treatment of . . xxi 218

of the swelling in . . xiv 14

nitrate of silver in simulated . xiv 13

nosological, position of . xxi 422

table of . . . xi 159

of breast, cure of an open . iii 557

M. Mericourt on . . xxii 159

suspended, case of . . xx 128

of eyelids, cure of . . iii 518

of kidneys, M. Rayer on . xv 490

of liver, Dr. Budd on . . xxi 59

of lungs, different forms of . xv 430

Dr. Stokes on . . xiv 242

frequency of . . . xv 430

physical signs of . . xv 434-8

secondary . . . xv 431

symptoms of . . xv 433

ulceration of . xv 432, 438

works on . . . xv 430

of the mediastinum . . xiv 243

of penis, case of . . . xiv 563

Lisfranc's delight on . xiv 12

of rectum, extirpation of . xi 198

M. Vidal on . . . xviii 452

of spinal column, cases of . xiv 53

of stomach, colour of skin in . xviii 210

complications in . . xviii 212

curious cause of . . xviii 209

diagnosis of . . . xviii 210

Dr. Barras on . . xviii 208

early symptoms of . . xviii 210

external . . . xxiv 274

hereditary . . . xviii 209

no hypochondria in . xviii 211

latent . . . . xix 27

pathognomonic sign of . xviii 212

remarks on . . . ix 477

remissions in . . xviii 212

second stage of . . xviii 211

third stage of . . . xviii 212

treatment of . . . xviii 212

yeast-plants in . . xxiii 510

of uterus, age most liable to . xviii 31

arsenic in . . . xvii 513

chalybeates in . . vi 95

discharge in . . . vi 95

Dr. Lever on . . . xvii 512

injections in . . . vi 96

invasion of . . . xviii 524

Lisfranc on . . . xviii 30

occult . . . . . xviii 31

relieved by leeches . . vi 96

statistics of . . . xvii 512

treatment of . . . vi 95

of womb, curability of . . xix 351

incipient stage of . . xiii 571

table of . . . . iv 372

on operating in . . . xiv 14

on quasi-epidemic . . . xix 27

potassa fusa in . . . xiv 13

primary and secondary . . xxi 429

physiology of . . . xxi 426

Cancer, rash and hopeless knife-work in xiv 13

relapse of, after excision . iii 65

after removal . . . xxi 436

relapses in, M. Duparcque on . ii 514

relative prevalence of . . vi 20

remarks on the treatment of . xiv 12

removal of . . . . xxi 435

by the knife . . . xi 165

returned, destruction of . . xiv 14

return of, after removal . . xi 166

signification of the term . . xxi 423

slumbering, in old age . . xiv 14

softening of . . . . xxiv 375

statistical tables of . . xi 165

starvation in . . . . xiv 15

superficial tubercles in . . xiv 14

swelling in, reduction of . xiv 14

three distinct species of . . xv 430

treatment after extirpation of . xiv 14

treatment of . . . . xxi 432

by ligature . . . ix 260

palliative . . . xxi 434

two distinct substances in . iii 382

use of plastic surgery in . . vii 412

uteri, nature of . . . vi 95

venous transmission of . . xi 163

Cancers least likely to return . . xiv 14

superficiality of some . . xiv 12

Cancerous affections, the blood in . xvii 432

cells, description of . . xxiv 374

diseases, Mr. Carmichael on iii 376, 382

matter, diagnosis of . . xxii 33

organization of . . iii 383

secondary development . . xi 164

tumour of face in the old . vii 144

ulceration of the larynx . . xviii 294

ulcer of the face in the old . xii 144

Cancrum oris, and stomatitis distinct xvii 297

description of . . . xvii 298

Dr. Hunt on . . . . xviii 367

mistaken for poisoning . . xviii 543

state of the vessels in . . xvii 298

Candles and lamps deteriorate air . xix 18

Canine madness in animals . . xxiv 90

Canker, water, Dr. Richter on . vii 471

Cannabis Indica, or hachish . . xxiii 217

efficacy of . . . xvi 357

uses of . . . . . x 225

sativa, medical properties of . xviii 368

Cannibalism in an Egyptian famine xix 534

Canstatt, Dr., his annual report . xvi 268

his special pathology, &c. xiii 327, 346

on the diseases of old age . xvii 100

Cantharides, a remedy in hydrophobia i 25

as an aphrodisiac . . . ii 549

camphor no antidote to . . ii 549

case of local action of . . xi 454

composition of . . . xiii 64

effects of, in some diseases . ii 550

green part only active . . ii 549

inefficacy of, in dropsy . . ii 549

in incontinence of urine . . ii 549

in paralysis of bladder . . ii 549

Cantharides, nature and action of . ii 548
nephritis rarely from . . viii 151
Cantharidin, a neutral substance . ii 549
experiments with . . . ii 549
not decomposed when absorbed ii 549
Cantharidis acetum . . . xiii 64
Cantharidum unguentum, Danish . xiii 65
Canula, with valve, for empyema . v 243
Caoutchouc threads for sutures . xii 282
Cape Coast command, mortality on xi 372
fatality of dysentery at . . xi 374
CAPELLO, M., on hydrophobia . iv 240
Cape of Good Hope colony, salubrity of xi 378
diseases of the . . xi 377
Town, topography of . . xi 377
Capillaries, arrestment of circulation in xiv 599
changes of the blood in . . viii 175
circulation in . . xiv 198, xx 157
circulation of blood in . xiv 575, 599
contractility of . . . xvii 572
Dr. Campbell on action of . xiv 183
form and arrangement of . xiv 286
function of, Mr. Jeffreys on . xvii 154
functions and powers of . viii 335, 338
functions of . . . . xiv 287
influence of cold on . . xvii 253
mechanical action of . . xiii 485
motionless blood in . . xiv 288
motion of blood in . viii 175, xviii 102
observations on . . . xxi 532
of the intestines . . . xii 529
on a propelling power in . iv 364
Schwann on the growth of . ix 518
stagnation of blood in . xvii 493
structure of . . . xiv 287, xix 244
transit of blood in . . . xiv 286
vermicular contraction of . xvii 154
Capillary attraction, experiments on xx 156
Matteucci on . . . xxiii 382
and nervous systems, relation of iii 12, 13, 16
circulation, cause of variations in xiv 600
in health . . . xvii 568
remarks on . . . v 336
velocity of . . . xix 262
blood-vessels, agency of . . xiii 162
distension, Dr. Billing on . v 202
power in the circulation . . vii 181
system, action of . . . viii 174
the chemical apparatus . xvi 146
vessels, anatomy of . . vi 218
growth of . . . ix 518
Capsules, supra-renal . . . vii 241
Caput obstipitum, two operations for xiii 4
Carbon, as an element of food . xvii 2
assimilation of, by plants . xi 437
elimination of, in the system . xiv 303
in vegetables, remarks on . iv 25
of plants, not from humus . xi 437
quantity required daily . . xvii 30
source of, in venous blood . iv 25
sources of, in the system . xiv 302-7
supplied by the bile . . xiv 303
Carbonate of ammonia, Dr. Philip on ii 146
Carbonate of iron in hooping-cough vi 222
Carbonization of animal matters xiii 186, 194
Carbonic acid and sugar, formulæ of xxiii 502
acid-asphyxia, the blood in . xvii 433
acid, death caused by . . ix 380
exhaled by the sexes . xvi 286
exhalation of . . xvii 257, xxii 189
fatal, though not inspired xvii 433
gas, asphyxia from . . xiv 197
in the blood . . viii 274
Mr. Parkin on . . iii 179
murder by . . vi 241
properties of solid . i 288
solidification of . . i 288
how far respirable . . ix 379
importance of, to life . viii 274
in air . . . . xix 13
in mineral waters . . xiv 326
of respiration . . . v 103
of the blood . . . i 590
on asphyxia from . . xii 264
quantity of, exhaled . xvi 285
oxide in air . . . . xix 14
Carbuncle, benign and malignant . x 350
inflammation in . . . x 349
malignant, in animals . . xiii 40
infection from . . xiii 44, 45
in man . . . . xiii 42
morbid anatomy of . . xiii 43
persons liable to . . xiii 45
rapid death in . . xiii 43
various forms of . . xiii 40
use of iodine in . . . viii 521
Carbuncular diseases, causes of . xiii 42
Carburet of sulphur in rheumatism ii 252
Carburetted hydrogen, poisoning by ix 380
Carcinoma, anatomical character of i 133
cell-theory of . . . xx 220
Dr. Carswell on . . . i 127
Dr. Hodgkin on . . . i 131
Dr. Macfarlane on . . . iii 558
in the blood-vessels . . i 129
of the breast, Mr. Mayo on . iii 65
of the lungs, cases of . . xx 90, 253
of stomach, external . . xxiv 274
of the thyroid gland . . xx 85
of uterus, case of . . . i 130
treatment of . . . xix 352
physical character of . . i 132
seat and origin of . . . i 129
signification of the term . . xxi 423
ulceration of . . . . i 134
uteri, statistics of . . . ix 442
table of . . . . iv 372
Carcinomata, on moveable . . xv 34
Carcinomatous matter, on injection of iii 383
Carcharadon, large jaw-bones of . xx 392
Cardiaca passio, Cælius Aurelianus on i 262
Cardiac affections, diagnosis of . xiv 241
and functional brain diseases . xxii 424
and gastric disease, case of . vii 127
arthritic diseases of old age . xvii 115
concretions in pneumonia . xiii 386

Cardiac disease affecting brain, treatment of . . . xxii 427

dropsy in, treatment of . vi 390

from distant affections . xxiii 179

from pericarditis . . xvi 328

from rheumatic diathesis . xvi 468

in insanity . . . xxii 425

jarring pulse in . . ix 485

inflammation, on returns of . xxiii 174

treatment of . . . xx 189

region, vibratory feeling over . i 448

sounds, direction and extent of i 484

with bronchial disease . . vi 390

Cardia, its nervous connexion . v 509

Cardialgia, on opium in . . . xxiv 142

Carditis and pericarditis, signs of . i 10

autopsy of a case of . . ix 434

case of partial . . . xx 223

case of rare form of . . ix 483

diagnostic symptoms of . . vi 134

M. Bouillaud and others on . ii 330

remarks on its diagnosis . ii 331

Caries and necrosis, Lisfranc on . xv 30

absorption of bone in . . xv 31

cauterization in . . . xv 31

in the ear, cause of . . iii 90

Lisfranc, on the cure of . . xv 31

of bones, nature of . . . xii 156

treatment of . . . xii 157

of the frontal bone, case of . xxi 471

or ulcerating osteitis . . ii 536

Sir C. Bell on . . . . vi 160

Carlisle, Sir A., his physiology . vi 189

on health . . . . vi 186

on vascular organization . xii 326

Carlsbad, Hufeland's saying on . xiv 343

mineral waters of . . . xiv 342

essays on . . . . . xix 371

waters in tic douloureux . vi 511

Carlyle, Thomas, on Scott and Cobbett . . . . . xviii 481

Carmichael, Mr., fallacious doctrines of . . . . . iii 377

his misconceptions . . . iii 381

on mercury in syphilis . . x 381

on tubercle and cancer . . iii 376

on venereal ulcers . . . vi 564

Carneæ columnæ, hypertrophy of . xii 457

Carnification of lungs in children . xiii 355

Carnivorous animals, nutrition in xiv 504, 515

Carolina, South (U.S.), medical college ii 591

Carotid arteries, ligature of . . xi 463

aneurisms . . . . . xxiv 421

arteries, effects of tying the . iii 154

artery, cases of ligature of . xxiv 323

case of aneurism of the . . v 610

case of tying the common . ix 436

common, case of ligature of . iii 522

on ligature of the . . xi 528

tying of the iii 266, xiv 132, v 463

tying, for tumour . . xiv 27

ligature of, in a child . . vi 233

tying the common, case of . v 610

cerebral disorders from . xxii 417

Carotids, compression of, as a remedy x 262

in mania . . . . xxii 14

effects of injecting foreign fluids into . . . . . i 558

effects on animals of tying . i 557

ligature of the . . . xiii 565

effects of . . . xxii 107, 109

on tying both . . . v 463

Carp, interesting encephalon of . xxiv 574

Carpenter, Dr., his general physiology xiii 160

his human physiology . xii 467, 486, xxiii 245

his manual of physiology . xxi 480

his microscopic report . . xv 259

his physiology . vi 590, vii 168, 185

his principles of physiology . xix 245

on animal physiology . . xvii 241

cells . . . . . xv 259

instinctive actions . . iv 530

life . . . . . xv 134

mental philosophy . . v 531

sensation . . . . v 531

structure of the brain . xxii 501

structure of shells xx 376, xxi 62, 76

the nervous system . . viii 506

vital phenomena . . vi 553

unity of function . . iv 532

white corpuscles . . xviii 590

Carpet-beating in Paris, clamour about v 456

Carpue, Mr., his plastic surgery vii 389, 392

Carpus, on dislocation of the . . vi 309

dislocation of, forwards . . x 51

Carrageen, or Irish moss . xiii 65, xvii 21

jelly of . . . . xili 66

with chocolate . . xiii 87

decoction of . . . xiii 66

Carswell, Dr., his illustrations of disease . . . . i 117, ii 465

his writings commended . . ii 465

on inflammation . vii 417, viii 169, 189

testimonial to . . . xi 269

Cartesian philosophers, arrogance of iii 12

Cartilage, structure of . . . i 231

articular, Mr. Liston's injection of . . . x 340

suppuration of . . xiv 545

vascularity of . . . x 339

the cells of . . . . ix 506

change in, from age . . ix 512

diseased, in scrofula . . xiii 169

diseases of . . . . xxi 131

fibrous, structure of . . xiv 267

ulceration of . . . x 346

loss of elasticity in . . xxi 132

of joints, inflammation of . xx 113

minute structure of . . ix 506

organical structure of . . xviii 86

permanent ossification of . xxiv 388

true, minute structure of . xiv 266

on ulceration of . . . xx 293

self-absorption of, remarks on . ii 164-6

ulceration of, Mr. Mayo on . x 345

the bone in . . . x 346

vascularity of . . xxi 131, 135

Cartilages, articular, diseases of . x 338

on vascularity of . . xxiii 71

vessels in . . . xi 452

of the bronchi . . . xii 332

independent morbid action of . x 340

loose, in knee-joint . . vi 171

of joints, diseases of . . x 330

on ulceration of . . ii 163

in mollusca, facts in . . xxi 547

morbid removal of . . . x 340

pathology of . . . . vii 535

removal of, Mr. Key's theory on x 341

reproduction of . . . xvi 504

solution of, in pus . . . x 341

ulcerated, granulations in x 344, 346

synovial growth in . . x 342

treatment of . . . xiii 171

ulceration . . . . . x 342

vitality of . . . . . xiii 169

Cartilaginous bodies in the pleura . iv 46

changes in the heart . . ii 326

transformations, on . . xxii 20

Caruncle of eye, diseases of . . xi 77

Carunculous growths in urethra . iii 395

Carus, Dr., his new cranioscopy . xiv 65, 75

his unworthy attacks on Gall . xiv 78

his wretched reasoning . . xiv 75

on development of the ovum . v 555

on the sex of the embryo . i 101

passage from a lecture of .

Cascade of acid water . . . viii 359

of Vinagre in Colombia . . viii 359

Casein, composition of . . . xvi 283

in flour of the cerealia . . xvi 283

in milk of the human female . xvi 283

in urine, doubt respecting . xi 336

solution of, by bile . . xvi 148

Case, medical, method of taking a . ii 476

ridiculous Italian medical . i 547

singular, of complicated disease i 250

Cases, by Dr. Forbes . . . xxi 539

defects in detailing medical . vii 122

fatal, two remarkable . . xxi 465

French, superiority of . . i 428

hospital, by Dr. Aldis . . i 490-2

medical, publication of . . xxiv 549

two kinds of . . . v 414

odd, by Sir George Lefevre . xix 241

Cashmere flora, by Mr. J. F. Royle i 215

Casper, Dr., his medical journal (German) iii 461

his statistics of diseases . . v 262

on hanging . . . . . v 615

on medical statistics . . xxiv 91

Castor oil, effects of, on the eye . xv 498-500

frictions in gout . . iv 243

Castrated animals, the cerebellum in xxii 541

Casts, anatomical, new . . . vii 278

Cat, domestic, temperature of . xii 142

Catalepsy, effect of civilization on . xviii 250

extraordinary case of . . xii 565

from lead poisoning . . x 457

insensibility to pain in . . xix 440

of thirteen years' standing . xx 263

Catalepsy, relation of to mesmerism xix 437

Cataleptic case, remarkable . xxiii 229-34

Catalogue of the Hunterian museum xv 195

of libraries, model for . . iii 476

Catalysis, a property of, in bodies . xi 143

Catalytic action in organic chemistry xi 144

Catamenia, age in relation to . . xiv 384

changes of ovary during . . xxi 89

distension of uterus by . . iv 373

effect of temperature on . xix 85

influence of on lactation . . xvii 541

in the male . . . . . vi 79

source of . . . . . vii 233

statistics of . . . . . xiv 382

suppressed, rheumatism from . xviii 522

tables of . . . . . i 103

Catamenial blood, acidity of . ii 274

Cataplasma conii, substitute for . iv 112

Cataplasms, on the application of . xv 37

Cataracta glaucomatosa, hopeless xvi 115

Cataract, accidents in operating for vi 57

an hereditary affection . . vi 52

on the use of belladonna in . xi 81

best condition of for operation xi 83

black, existence of . . . . xvii 329

blindness after operation for . vi 58

colour of . . . . . vi 54

combined operation for . xi 86

commencement of . . . vi 52

depression of . . iv 258, xi 84

by Dr. Brett . . . xxiv 367

diagnosis in, Mr. Tyrrell on . xi 80

difficulties in extraction of . xi 82

displacement of . . . iv 257

Mr. Scott on . . . xvii 341

division into four classes . vi 54

division of . . . . . iv 257

through sclerotica . . xvii 344

Dr. Jacob on . . xvii 343

atrophy of eye from . xvii 236

through cornea . . xvii 343

double, peculiarity of . . vi 53

Dr. Brett on . . . . xxiv 364

dressing after operating in . vi 56

drilling of . . . . . xi 82, 85

duration of . . . . . vi 55

error in regard to . . . xi 80

errors in operating for . . vi 57

external lateral section in . v 35

extraction of . . . iv 257, v 285

affections after . . xvii 339

bad success of . . xvii 331

hemorrhage in . . xi 83

from glaucoma . . . xviii 55

glasses, remarks on . . xvii 344

influence of age on . . vi 51

in one eye, operating in . . xxiii 374

remarks on . . . vi 52

knives, straight and curved xvii 334-7

medical treatment in . . xxiv 364

membranous, operating in . vi 58

Mr. Charles Guthrie on . . xx 512

Mr. Jeaffreson on, criticised xviii 416-19

Cataract, M. Maunoir on . . vi 50

Mr. Morgan's operation for . xvi 326

M. Roux's mode of operating in vi 56

Mr. Scott on . . . . . xvii 329

knife for, figure of . . xvii 331

Mr. Stevenson on . . . iii 207

Mr. Tyrrell on . . . . xi 79

needle for . . . . xi 85

on extraction of . . xi 82

on operations for . . xi 81

muscæ volitantes in . . xi 80

new mode of distinguishing . vi 231

of extraction of . . xv 245

new treatment of . . . v 604

of twenty-three years, cured . xv 152

operation for, prognosis of . vi 58

for solution of . . xi 86

of breaking up . . xi 84

on operations for . . . v 285

posture in operating for . . xi 82

reclination of the . . . iv 258

results of operations for . . vi 58

Roux's operations for . . x 38

season for operating for . . xvii 235

section of the cornea in . xvii 335, 337

single and double . . . vi 53

sincipital combustion in . . vii 64

Sir D. Brewster on . . v 602

site of opacity in . . . v 44

solution of . . . . . xvii 343

spontaneous cure of . . vi 53

spots in, errors respecting . vi 54

superior section in . . . v 35

three sections of cornea for . xi 82

treatment of, after operation . xi 83

Catarrh, abstinence from liquids in ix 488

causes of varieties of . . xi 404

epidemic, Dr. Craigie on . iii 455

in relation to phthisis . . ix 329

nasal, ciliary epithelia in . xvii 520

no nasal mucus in . . . xvi 151

of bladder, injections in . iii 521

use of caustic in . . vi 565

pituitous and dry, combined . iv 301

vesical, Civiale's practice in . i 497

Catarrhal affections of animals . xxiv 85

rhonchi, remarks on . . iv 302

Catarrhe, suffocant aigu . . xii 357

Catechu, Dr. O'Shaughnessy on . xvi 358

tincture of, in sore nipple . xiv 582

Caterpillar, drowned, recovery of . xxi 270

Catgut as a ligature for arteries . xxii 91

ligatures for arteries . . xiv 24

Cathartics, bad effects of frequent . viii 496

in hydrocephalus . . . i 20

Catherine II, inoculated for smallpox i 605

CATHERWOOD, Dr., on chest-diseases xiii 220

Catheter, double current, of Hales . xii 398

for nasal duct, figure of . . xxi 311

nasal, of Gensoul, described . iii 449

new mode of introducing . xi 535

prostate, form and length of . v 467

Catheterism, history of . . . xvi 56

Catheterism, Mr. Cock on . . xii 346

Catheterizing the female bladder . iii 423

CATLOW, Mr., his mode of causing sleep xix 476

Cat's eye, amaurotic . . . x 38

tail plant, down of, in burns . i 580

Cattle, poisonous, in America . xviii 555

Caucasian and Mongolian races . xxiv 454

or Georgian race, the proper . xxiv 465

Cauda equina, structure of . . vii 503

tumour in . . . . . xiv 396

Caudate cells in cancer . . . xxiv 375

Cauliform excrescence of os uteri . xxiv 511

Cauler Zeree, compound powder of xix 75

Causes, concurrence of, in disease xxiii 43, 59

of disease, their importance . vii 120

of things, Aristotle on . . xv 444

Caustic, discrimination in the use of xxii 368

lunar, into larynx . . . xxiv 501

Caustics to the scalp, caution on . xxii 172

use of counter-agents of . xxii 171

Cauterization of cervix uteri . . xiv 235

Cautery, actual, little pain from . xxii 397

to spine, in leucorrhœa . xxiii 293

observations on . . xxii 172

CAUTLEY, Captain, his fossil zoology xxi 476

Cava, inferior, anomaly of . . xviii 224

Cavalry horses, anecdote of . . xix 309

Caverns, or excavations in the lungs xxiii 431

Cavities, accidental articular, close xviii 82

close, on diseases of . . xviii 84

functions of . . . xviii 83

ganglial . . . . . xviii 83

injection of tincture of iodine into . . xviii 88

obliteration of . . xviii 87

pathology of . . . xviii 79

serous surfaces of . . xviii 80

morbid, close, cellular . . xviii 82

glandular . xviii 82

of the heart, changed dimensions of ii 338

size of, unequal . i 432

on accidental cellular . . xviii 82

serous, accidental, close . xviii 81

synovial, M. Velpeau on . xviii 81, 85

Cavum cranii in human races . viii 378

CAZAUVIEILH, M., on suicide, &c. x 129, 162

CAZENAVE and SCHEDEL on skindiseases . . . xv 372, 399

CAZENAVE, M., his manual, piracy on xxiii 372

on syphilitic eruptions . . xxiii 345

on diseases of the skin . . xxi 491

CEELEY, Mr., his variolation of cows ix 81

on variolæ vaccinæ . . x 464, xiv 397

Celestial intercourse, case of . . xix 471

Cell development, theory of . . xxii 262

growth, phenomena of . . xxiii 379

life in animals, Dr. Carpenter on xv 261

in plants, and the egg . xv 270

the primary, changes of . . xxiii 138

formation of . . . xxiii 137

Cells, animal, growth of . . ix 505

and globules, Baumgaertner on xxiv 135

and organisms, life of . . xx 71

Cells, anatomy of . . . . xix 566

animal, functions of . . xv 261

cancerous, description of . xxiv 374

contagious, inoculation by . xx 214

development of in animals . xv 263

nutrition in . . . xix 565

epithelial, development of . xv 278

independent vitality of single ix 524

in plants, formation of . ix 498

metabolic phenomena in organic ix 523

moving molecules within . xx 121

Mr. Addison on the structure of xvii 94

on the multiplication of . . xv 270

of plants, changes in . . ix 501

changes of form in . . ix 503

double life of . . . ix 501

multiplication of . . xxiv 223

rotatory motions in . xix 361

observations on . . . xxi 422

on the origin and function of xv 259

of the liver . . . . xviii 339

of the bones, observations on . xx 306

on the pathology of . . xviii 339

plastic phenomena in organic . ix 522

points in morphology of organic ix 522

on the production of living . xix 168

progressive metamorphosis of xx 72

regressive metamorphosis of . xx 73

secreting, development of . xx 294

on the transient existence of . xv 269

vegetable, functions of . . xv 259

Cellular and fibrous tissues . . xiv 272

cavities, pathology of . . xviii 82

form of animal tissues . . xiii 164

formation and cystic germ . xiii 339

membrane, erysipelas of . ii 23

substance, nervous energy of . ix 408

properties of . . iv 170

remarks on . . . xxi 494

tissue, elements of . . ix 514

formation of . . . xviii 273

induration of in infants ii 361, xx 553

Cellulitis venenata, Dr. Williams on xiii 95

Celosomi, monsters so called . viii 31

Celsus lithotomy revived by Dupuytren ii 99

Mr. Lee's translation of . iii 204

Cementum, or crusta petrosa of teeth xx 380

Cemeteries, on arsenic in . . xiii 196

distance of, from towns . xv 517

inspectors of, proposed . xv 523

observations on . . xv 520, 522

proper extent of . . . xv 521

Centipede, heart and arteries of . xx 498

Central heat, Mr. Taylor on . xxii 229

Centralization, observations on . xix 139

Central nervous influence, what . iii 209

Centres of nutrition, Mr. Goodsir on xx 290

Centrum vitale, the . . . i 560

Cephalalgia, cured by ol. terebinthinæ xii 432

nervous, treatment of . . xvi 362

seven forms of . . . xii 104

varieties of . . . . i 24

Cephalhæmatoma . . . . i 182

Cephalhæmatoma, anatomy of . vii 89

characters of . . . vii 87

diagnosis of . . . . xiii 181

diverse opinions on . . xiii 180

Dr. Mauthner on . . xxi 384

etiology of . . . . vii 89, 90

frequency of . . . vii 88

Mr. Close on . . . xxiii 300

neconatorum . . . xxi 458

of infants . . . . vii 86

origin of . . . . xiii 181

period of origin of . . vii 87

prognosis in . . . vii 91

progress of . . . . vii 87

pulsation in . . . vii 87

report on . . . xvii 549, xx 552

sub-aponeurotic . . . vii 86

sub-pericranial . . . vii 86, 87

super-meningeal . . . vii 86

termination of . . . vii 91

three species of . . . xiii 180

the blood in . . . vii 90

the osseous ring in . . vii 87, 89

treatment of . . . vii 91

with fracture, case of . . xxiii 413

Cephalhæmatomatous infants' mothers vii 188

Cephalic bellows-sound . . vii 267

fever in children . . xxi 383

sound of deglutition . . vii 266

respiration . . . vii 266

the heart . . . vii 266

the voice . . . vii 266

version, Dr. Ryan's mistake on ii 525

rarely practicable . . ii 525

Cephaloma, Dr. Carswell on . . i 128

Cephalo-rachidian fluid, use of . xxii 413

Cephaloscope, Mr. Curtis on the xiii 509, 511

Cephalo-spinal fluid, Dr. Nasse on the iv 436

Cephalotribe of M. Baudelocque . xiv 371

on the use of the . . xix 417, 419

Cephalotripsy in deformed pelvis . xix 417

practice of . . . . xii 489

Cereal grains, proximate principles of xvii 19

Cerebellum absent when cerebrum is so xxii 530

cancer of the . . . xxii 310

case of abscess of the . . v 238

comparative anatomy of . xxii 535

experiments on removal of xxii 530, 532

functions of the viii 576, xii 483, xvi 368

in castrated animals . . xxii 541

its sexual function . . xxii 534

Mr. Solly's discoveries on . xxiv 575

organic diseases of . . iii 315

pathology in relation to . xxii 542

restraining power of . . xvii 403

special function of the . . xxii 529

structure of . . . xix 578

tumours in the . . . iii 221

Cerebral and sympathetic connexion xxii 406

affection in abdominal disease ix 475

affections, causes of . . iii 318

cold affusion in . . xxii 398

from lead . . . x 454

Cerebral affection, from pericarditis xii 333

affections, Mr. Mayo on . v 386

on bleeding in . . viii 489

rare in Hindoos . . xvii 155

analogues in invertebrata . xxii 499

aneurism . . . . xxiv 418

auscultation . . vii 265, xx 255

circulation, disorders of . xxii 408

influence of posture on . xxii 410

obstruction of . . xxii 415

variations of . . xii 99-100

congestion, active . . xii 100-1

in children . . . xxi 382

in corpses, caution on . xxii 411

passive . . . . xii 100-1

determination, Dr. Hope on . xii 100-1

diarrhœa and vomiting . . ix 475

observations on . . xvi 236

disease, and disease of kidneys x 586

after scarlatina . . iii 309

incision of scalp in . vi 381

in children . . . xv 325

in infants, convulsions from xx 364

organic, symptoms of . iii 316

diseases, arrangement of . iii 303

causes of . . . xx 33

Dr. Abercrombie on . iii 301

general division of . iii 303

long issue of scalp in . xvii 96

disorders, from tying carotids . xxii 417

disturbance, the tongue in . v 402

effusion, new light on . . xi 153

on coma from . . xxii 405

excitement in fever, respiration in xvi 236

forms in higher vertebrata . xxii 500

hemorrhage, &c. case of . xxii 387

in children . . . xvii 301

in newborn infants . vii 92

its relation to paralysis . i 142

observations on . . xx 35

remarks on . xxii 389, 395, 405

valuable case of . . i 143

hernia, case of . . . ix 221

treatment of . . . ix 221

inflammation, bleeding in . iii 307-8

causes of . . . iii 309

emetic tartar in . . i 421

forms of . . . iii 305-7

in fever, cold dash for . ii 64

mercury in . . iii 308

purging in . . iii 308

treatment of . . iii 307

with convulsion . . iii 306

irritation, treatment of . . xx 36

matter, analysis of, by M. John i 288

injected into veins . viii 325

meninges, cancer of . . xxii 310

meningitis, treatment of . xxii 461

motion, lesions of . . xx 28

murmur, Dr. Smyth on . iv 536

nerves, Mr. Grainger on the v 498-9, 593

origin and continuation of xviii 141

their continuity with others ii 135

Cerebral nerves, non-congestion in hanging . . . . xxii 412

nosology, difficulties of . . iii 301

œdema, M. Pinel on . . xx 23

treatment of . . . xx 38

paralysis, irritability in . . xi 454

pathology, M. S. Pinel on . xx 18

physiology, Mr. Noble on . xxii 488

powers, operations of, on the bodily machines . . xxiv 194

pressure, on normal . . xxii 413

softening, M. Pinel on . . xx 35

colours of . . . xvii 529

two kinds of . . . xvii 529

substance, peculiar change of . xiv 225

symptoms in fever, preventing xvi 235

intermission of . . xxii 416

system, Dr. Hall on the . xvi 163

in simpler animals . ix 100

tissue, softening of, Dr. Gross on xxiii 72

tumour, remarkable case of . xix 376

vessels, how protected . . xxiii 142

relative fulness of . . xvii 127

solid deposit in the . iii 70

unalterable fulness of . xvii 127

vomiting, observations on . xvi 236

with renal disease . . ix 370

Cerebritis and meningitis, diagnosis of xii 101

Cerebritis, chronic, on curing . xii 103

Cerebro-spinal—arachnitis, report on xxiii 302

diseases of infants . . xi 208

fibrils . . . xvii 383, 386

fluid, admixture of . xiv 468

analysis of . . xiv 466

confluences of . . xiv 464

decrease of . . xiv 468

in animals . . xiv 468

increase of . . xiv 467

Magendie on . . xiv 463

movements of . . xiv 466

quantity of . . xiv 466

report on . . xxii 277

seat of . . . xiv 463

uses of . . . xiv 466

irritation, causes of . . xix 101

membranes and vessels . xxii 278

nerves, structure of . vii 501

nervous fibres . . xiv 277

phenomena . . . vii 228

ventricular fluid . . xi 153

with cardiac disease . xxii 425

Cerebrum, Sir C. Bell on . . ix 126

and sensory ganglia, on . . xxii 504

effects of removal of . . xxii 508

general functions of . . xxii 511

M. Foville's anatomy of . . xviii 432

operations of the . . . xvi 159

structure of . . . . xviii 435

unity of . . . . xvi 367

Cerise, Dr., his work on education xiv 527

Certificate, to females, from M. Ricord xii 365

medical, remuneration for . x 218

to assurance societies . . v 311

Ceruse manufactories, on colic in . ii 573-6
precautions in . . ii 574
workmen in, advice to . ii 575
manufacturers, advice to . ii 575
Cervical nerves, origin of . . i 550
vertebræ, dislocation of . xiii 266, xiv 31
Cervex uteri, affections of, Lisfranc on xviii 28
amputation of, Duparcque on ii 517
case of imperforate . . xvii 542
cauterization of . . xiv 235
during pregnancy . . xvi 185
efficacy of leeches to the . ii 516
excision of, by Lisfranc . i 496
fleshy growths on the . ii 515
hypertrophy of . . xix 356
inflamed, treatment of . vi 90
inflammation of . . v 89, xix 347
leeches to inflamed . . v 90
preternatural redness of . xviii 28
ulceration of . . . xvii 546
Cetaceous tribes, on the diffusion of iii 369
Cetraria Islandica (Iceland moss) . xiii 66
Cetrarin, the principle of Iceland moss . . . . . . xiii 67
use of, in intermittents . . xiii 67
Ceylon, aboriginal population of . xxiv 462
climate of . . . . xv 3
continued fever in . . . xv 4
description of . . . xv 2
great fatality of fever in . . xv 4
intermittent fever in . . xv 4
medical reports on . . xv 1
mortality of troops in . . xv 4
non-restraint of lunatics in . xix 608
remittent fever in . . . xv 4
smallpox and vaccination in . vii 527
CHADWICK, Mr., his sanitary reports . . . xv 286, 328, 346
on interments in towns . . xvii 403
on duration of life . . xviii 197, 207
Chaff and bark used as food . . xvii 16
CHAILLY, M., on midwifery . xiv 366, xix 416
praise of . . . . xiv 373
Chair, Mr. Curtis's acoustic v 404, xiii 512
Chalazion of the eyelid . . . xviii 420
Chalk-stones, new remedy for . xiii 264
Chalybeates in scrofula, value of . xi 209
Chalybeate water, formula for artificial ii 555
waters . . . . xiv 326
cases benefited by . . xiv 338
CHAMBERLAYNE, Dr. P., and the Czar of Russia . . . i 601
CHAMBERS, Dr., letter to . . vii 534
CHAMBERS, Mr. R., his memoir on Dr. A. Combe . . . xxiv 581
CHAMPIONNIERE, M., on syphilis . x 381, 418
Channel Islands, climate of . . xii 169
Chantiers d'Ecarrissage of Paris (Appendix to vol.) xi 38
Chancre, against cauterization of . xxiii 349
Dr. Colles on . . . v 11
effect of mercury on . . v 23
indurated primary . . x 404
Chancre, induration of cicatrix of . x 405
nature of . . . . x 382
phagedenic, tinct. of iodine in . xii 251
Sir C. Bell on the cure of . vi 171
solution of tannin used for . x 403
treatment of . . . . v 23
Chancres, aphthous, Mr. Key on . xii 336
aromatic wine used for . . x 402
causes of varieties of . . xii 336
in the female, remarks on . x 411
indurated, healed without mercury xii 338
larvés of French surgeons . xii 367
local treatment of . . . x 401
mercury in . . . x 398, xii 337
Mr. Key on . . . . xii 335
nitrate of silver in . . . x 401
opiate cerate used for . . x 402
phagedenic, treatment of xii 339, 340
tincture of iodine in . . viii 523
treatment of . . . . xii 337
two stages of . . . x 382
varieties of indurated . . xii 338
CHAPELLE, Mde. La, on ergot of rye ii 525
CHAPMAN, Dr., on diseases of viscera xix 21
on eruptive fevers, &c. . . xxi 358
CHARAKA on diseases . . . xxiii 540
the Indian medical writer xxiii 526, 530
Charbon essentiel of Chabert . xiii 41
or malignant carbuncle . . x 350
symptomatique of Chabert . xiii 41
Charcoal, case of morbid appetite for iv 222
in cholera and dysentery . ix 74
Charcoal fumes, toxic principles of . ix 377
vapours, poisoning by . . ix 377
Charenton, effect of restraint in . xix 597
former exhibitions at . . xix 601
government of . . . xix 600
la Maison Royale de . . i 272
house-pupils in . . . xix 600
lunatic asylum, account of . xix 594
Charitable institutions in Denmark iii 572-5
Charité, l'hopital de la, account of i 281
Charities, Irish medical, Mr. Phelan on ii 183
medical, of Ireland . . xiii 513
Charity, private and public, remarks on xxii 339
CHARLTON, Dr., on scarlatina in Newcastle . . . . . xxiv 286
Charter, new, for general practitioners xix 614
of the College of Surgeons xvii 279
remarks on . . xvii 280
of the College of Physicians . xix 552
of the London University . iii 293
CHASE, Dr., his femoral truss . v 305
his inguinal truss . . . v 301
on the radical cure of hernia . vi 341
his trusses for hernia . . vi 344
his ventro-inguinal truss . v 304
Chastity in medical language . xxi 446
Chatham, museum of . . . vii 224
Chaudes, spa at . . . . . xiv 353
CHAVASSE, Dr., his treatment of laryngitis . . . . xviii 290
on secale cornutum . . iii 164

CHAZAL, M., his Atlas of embryogeny ix 2

Cheek, plastic operations on . xix 403, xxi 305

remedying defects of . . vii 410

Cheese, decayed, on death from the use of . . . . . xxiii 161

Cheiloplastic, etymology of . . viii 407

operation . . . . . vii 407

of Dieffenbach . . vii 408

CHELIUS, Dr., his Manual of Surgery xi 305-6, xx 110, 119

his surgical observations . ii 259

on ophthalmic medicine . . x 3, 21

Cheltenham, a medical dinner at . iv 558

medical topography of . . vi 384

waters of . . . . . xiv 345

Chemical affinity and capillary attraction . . . . . xxiii 384

analysis, defence of . . xxiii 513

Dr. Fresenius on . . xvii 244

improvement in . . xi 134

Mr. Parnell on . . xv 531

and organic combination . xx 71

and vital force, action of . xiv 497

composition of the body xix 249, xxi 541

change, effect of life on . xxi 493

domain, limitation of . . xiv 494

formulæ, remarks on . . xvi 478

manipulation, Mr. Faraday's . xv 538

pathology, caution on . . xvii 449

pharmaceutical errors . . xvi 203

remedies, Dr. Paris on . . xvi 195

on the new . . . xix 374

school of physiology . . i 383

science, limits of . . . xv 142

prospects of . . . vii 174

tests, manual of . . . vii 533

therapeutics, remarks on . xv 500

Chemist, peculiar privilege of the . xii 7

source of his power . . xii 6

Chemists, connexion of physicians with x 220

young, friendly advice to . xv 512

Chemistry, Animal, Liebig on . xiv 492, 521

built on calculation . . xii 6

Dr. Kane's Elements of . . xi 514

Dr. Turner's, by Liebig . . xxiii 584

elements of, by Dr. Turner . v 216

future prospects in . . . xi 149

in relation to medical studies . x 185

to theology . . . xviii 531

its connexion with meteorology xxi 118

its rank as a science . . xxi 110

its relation to medicine . . xvi 145

medico-legal, Gusserow on . v 212

Mitscherlich's elements of . ii 188

treatise on . . . vi 505

Mr. Ede's facts in . . . iv 498

Mr. Fowne's Manual of . xix 245

of Food, Liebig on . . xxiv 264

of the ancient Hindoos . . xxiii 535

of the Four Seasons . . xxiii 238

on comparative animal . . xvii 425

organic . . . . . xi 436

Dr. Paine on . . . xv 144

Dumas and Boussingault on xviii 531

Chemistry, organic, introduction to xviii 531

pathological, difficulties in . xvii 447

remarks on . . . xvii 447

treatises on . . . xvii 424

Dr. Bird on . . . xiv 576

value of . . . . xvii 424

essay on . . . . xix 364

place of, in medical study . xi 148

on physiological . . . xvii 425

Professor Liebig's letters on . xvii 224

progress and state of . . xi 129

proper mode of teaching . xi 149

remarks on the science of . v 323

retrospective glance at . . vii 144

treatises on . . . xi 129

utility of the study of . . ix 425

Chemosed conjunctiva, dividing . vii 156

Chenopodium olidum, emmenagogue vii 235

Chenouk Indian, account of a clever x 483

Cherry-laurel water, in neuralgia . i 263

CHESELDEN, eulogies on, fatality of xxiv 368

Chest, Andral on diseases of the . iv 285

deformities of, from scrofula . iii 172

Mr. Coulson on . . iii 169

treatment of . . . iii 173

development of the . . v 407

diagnosis from deformities of . iii 226

diseases dilating one side of . xix 110

diseases of the . . . ix 484, 217

Dr. Gerhard on . . iii 185

Dr. Phillipp on . . iii 184

Dr. Stokes, his callipers for the v 425

on diseases of the iv 285, 324, v 423

effect of passions on the . v 136

effusion in, three sub-stages of xiii 365

estimate of its dimensions . vii 356

-explorator, Dr. Babington's xviii 343

fitness of, for physical signs . iv 289

general heteromorphisms of . vii 361

impediments to sounds of the iv 294

in phthisis, expansion of, early i 480

inspection and mensuration of vii 353, 355

instrument for measuring . v 425

measurement of, by the eye . iv 322

momentary changes of form of vii 354

narrowness of, as to phthisis . vii 368

normal and altered forms of . vii 357

observations on sounds in . xiv 176

particular heteromorphisms of vii 365

pathological forms of . . vii 363

physiological forms of . . vii 354-63

prominences of, M. Woillez on vi 34

quantity of air contained in . xxiv 327

sounds of, in health and disease iii 545-6

statics of the . . . xvii 152

CHEVALLIER, M., on calculus . xii 388

on colica pictonum . . ii 573

on diseases of artisans . . i 282

of cutlers . . . v 257

of printers . . . i 282

CHEVERS, Dr., on acute aortitis . xiv 127

on death after operations . xviii 333

diseases of the aorta . . xvi 323

the coronary arteries . . xviii 334

CHEYNE, Dr., his remarks on empirics xiv 426

CHEYNE, Dr. John, his life and works i 614

eulogy on . . . . i 615

record of his death . . i 614

CHEYNE, Dr. George, a teetotaller . xix 124

CHEYNE, Dr., of Dublin, on his writings . . . . . xviii 56

CHIAPPA, Dr. Del, his clinical report . . . . . i 249

interesting case by . . . i 250

on nervous diseases . . i 252

on sthenic diseases . . . i 250

Chicane and deception, on medical xxi 451

Chick, air in the evolution of the x 229

Chicken-breast, cause of . . v 139

Dupuytren's treatment of iii 173

emphysema from . . xvii 296

mode of production of . xvii 294

Mr. Coulson on . . iii 173

respiratory movements in xvii 295

treatment of . . . xii 181

Chicory, wholesomeness of . . xvii 21

Chilblains, use of creosote in . xiii 69

iodine in . . . viii 522

nitrate of silver in . . xiii 62

Child, accidental death of, at birth vii 139

apoplexy of, from retained head ii 360

arm of, amputation of in labour ii 526

birth of living, on 179th day . xiv 256

causes of death of, in labour . vii 139

-crowing of Gooch . . . ii 113

death of in womb, remarks on v 179

enormous weight of at birth . vii 586

head of, injury to in labour . xiii 548

-murder, case of alleged . . xiii 250

newborn, proof of life in . ix 45

poisoned by laudanum, case of xxiii 299

relation of its sex to parturition xix 541

respiration of during birth . iv 101

restoration of a stillborn . viii 596

retention of its head in labour ii 360

signs of live birth in a dead . vi 63

stomach of, different from adult ii 539, 541

figure of . . . ii 539

surviving loss of brain . . ii 360

surviving perforated head . ii 360

death, auscultic diagnosis of . i 96

death, before labour, signs of i 111

during labour, signs of . i 111

foot, in labour, diagnosis of . i 115

Childbed fever, Dr. Litzmann on . xxi 275

deaths in, in England . . ix 355

diseases of women in . . xiii 100

Childhood and infancy, on diet in . xxiii 299

diseases of . . . . . xxiii 302

report on . . . . xx 554

CHILD, Dr., on indigestion, &c. . xxiii 577

CHILDS, Mr., his splendid peroration xiii 221

on spinal curvature . . xiii 221

Children, administration of mercury to xxii 169

American management of . v 412

anatomical peculiarities of . ii 354

artificial food to, thrush from xxiv 431

at the breast, on fever in . xx 359

Children, born alive in 240 twin cases ii 72

born in France, table of . xiii 294

bronchial phthisis in . . xvii 305

respiration in . . xii 358

causes of desertion of . . xiii 297

cerebral affections of . . xii 346

hemorrhage in . . xvii 301

tubercles in . . . vii 576

chronic peritonitis in . . xxiv 47

confining, evil effects of . ii 386

on convulsions in . . . xxi 391

treatment of . . xxi 392

contraction of limbs in . . xxi 471

cramming, bad effects of . ii 383

crowing disease in, cause of . iii 29

cry of, in diagnosis . . ii 356

desertion of in France . . xiii 298

development of, Vogel on . ii 355

dietaries for . . . xvii 48, 51

dietetic rules for . . . ii 386

diet of . . . . . vii 382

diseased, on round stomach in ii 541

diseases of . . . . . xxii 548

diagnosis in . . . vi 426

Dr. Doepp on . . xxi 407

Drs. Evanson and Maunsell on iii 439

Dr. Rees on . . . xviii 236

Dr. Stewart on . . xiii 222

Mr. Rees on . . xiii 219

of the brain of . . xxi 381

report on . . . xxiii 297

treatises on . . ii 353, xv 320

dosing, with opiates . . xxi 355

Dr. Evanson on diseases of . xi 198, 210

Dr. Maunsell on clothing of . iii 442

on diseases of . . xi 198, 210

Dr. West on the pneumonia of xv 543

dropsy in, Dr. Wolff on . xiv 202

dyspepsia of . . . . xii 165

effect of civilization on . . xviii 241

eruptions on heads of . . xxiii 509

Esquimaux mode of cramming ii 383

evils of table dainties to . x 527

facial hemiplegia of . . x 269

food and clothing of . . vi 188

formulæ for diseases of . . vi 516

gin-sling and milk-punch to . v 412

hardening of, remarks on . ii 126

health of, in cotton mills . xviii 147

Sir A. Carlisle on . . vi 189

heat of, at birth . . . iii 440

illegitimate, statistics of . vii 130

irritability and sensibility of . ii 355

management of . . . . iii 442

maternal management of . xi 224

mature, site of umbilicus in . xvi 310

mesenteric phthisis of . . xvii 311

mortality of . . . vii 293

in Austria . . . xxii 344

in London . . . vi 187

M. Villermé on . . iii 52

Mr. Combe's advice on . xv 58

Mr. Dendy on diseases of . vi 426

nervous tremor of . . . vii 583

Children, newborn, number of deaths in 16,654 . . . ii 74
of 13-15 lb. weight . ii 79
number of stillborn, in 16,654 ii 72
in 16,434 labours . ii 72
of the uneducated, dullness of vii 383
on much medicine to . . ii 358
overtasking the minds of . ii 386
pathology of the brain of . viii 489
phthisis of . . . . . xx 97
physical education of . . xx 357
physiognomy of, in diagnosis . ii 356
plurality of, more dangerous than single . . . . . ii 90
plurality of, report on . . xx 529
pneumonia and phthisis of . xvii 310
poisoning of, by opium . . xx 332
precocious development in . viii 19
premature, in 1121 stillborn ii 72
pulmonary phthisis in . . xvii 310
quinine in diseases of . . xiv 560
report on stillborn . . xxi 352
on the diseases of. . xvii 549
skull of, in diagnosis . . ii 357
softening of intestinal mucous membrane of . . . ii 551
stillborn, remarks on . . viii 52
stomacace in, Dr. Wendt on . xiv 203
temperature of . . . . xix 266
the cerebro-spinal system of . iii 440
thrush in . . . . . xxiv 422
treatise on diseases of xvii 293, xx 205, xxiii 237
treatment of fractures in . xii 544
tubercular meningitis in . . xvi 445
tuberculous disease in . . xvii 304
twins, in 16,434 labours . . ii 72
unnatural management of . ii 385
valvular murmur in . . xxiii 180
variableness of the pulse of . viii 367
voice of, in cerebral disease . iii 221
weaning of, remarks on . . ii 386
weight of brain of . . xxi 387
wine, &c., injurious to most . ii 389
Children's diseases, essays on . xiv 202
hospital in St. Petersburg . xxi 470
dress, remarks on . . ii 126
Chilognatha, development of . xx 491
structure of the . . . xx 489
Chiloplastic operations . . xxi 303
Chilopoda, structure of the . . xx 489
Chimney-sweeper's cancer . . xvii 87
effect of civilization on . xviii 253
Chimneys, ventilation by . . xix 7
Chimney-ventilator, a cheap . . xviii 501
Chimpansé, blood-vessels of the . xvi 380
comparative anatomy of the xvi 373, 381
extremities of the . . xiii 474
muscular system of the . xvi 378-380
neurology of the . . . xvi 380
splanchnology of the . . xvi 380
China, establishment of hospitals in xii 290
China, diseases in the navy in . xxii 180
diseases of the natives in . xxii 186
Dr. Wilson's Medical Notes on xxii 179
medical missions to, and in iv 568, xiv 216
Chinese, Korean, and Japanese, races xxiv 462
midwifery case . . . xiii 569
monster A-Ke . . . xii 376
Chiretta (gentiana chiretta), in ague iii 348
Chiroplasty, observations on . xix 397
Chirosophus, the, his book . viii 69, 71
Chirurgeon, a famous Thuringian . xiv 405
Chirurgeons, history of . . xiv 405
of the long and short robe xiv 405, 406
Chirurgi physici, history of . . xiv 405
Chittick, Dr., his remedy for stone xii 391
Chloracetic acid, properties of . xi 148
Chloride of lime, not disinfectant . xiii 62
use of . . . . xiii 62
in wounds . . . v 575
of silver, use of . . . xii 567
of soda, in intermittent fever . ii 553
in typhoid fever . . ii 64
qualities of . . . v 564
of sodium, in the blood . xxiv 270
Chlorides, in typhoid fever, Chomel on ii 64-8
of lime and soda, in ozœna . i 270
used in fever by Dr. Reid, 1826 ii 64
Chlorine, as an element of food . xvii 4
in bronchitis . . . vi 533
its action on the system . iv 215
mode of inhaling . . . xx 449
Chlorine fumigations in cholera . i 15
gas, cases not benefited by . iv 215
injection of in hydrocele . ii 267
in ulcers of lungs . . iv 215
use of in phthisis . . iv 212
inhalations, in bronchitis . iv 224
Chlorophyle, properties of . . xvii 20
Chlorosis, ammonial injection in . xi 178
analysis of the blood in . . vi 447
and diseased spleen, analogy of iii 346
cause of in the blood . . iii 157
complexion in, diagnosis of . xiii 332
complicated . . . . xi 175
complications of . . . . xi 177
definition of . . . xi 175
Dr. Ashwell on . . . iii 156
Dr. Balbirnie on . . . iii 177
Dr. Bland's pills for . . viii 252
Dr. Canstatt on . . . xiii 332
etiology of . . . . . xi 176
incipient form of . . . xi 175
in women, perfectly regular . xiv 393
M. Bland's remedy for . . iii 177
menorrhagic, M. Trousseau on viii 250
use of iron in . . viii 250
mischief of bleeding in . . xi 177
Mr. Fox on the cause of viii 542, 543
new theory of . . . xvi 227
not an inflammatory disease . xi 176
observations on . . xiv 389, 392
pathology of . . . xiv 393

Chlorosis, sthenic species of . . xiv 593

the blood-globules in . . xvii 141

time for emmenagogues in . xi 178

treatment of xi 177, xiii 333, xiv 394, xix 346, xxi 89

with hematemesis . . . . xix 345

Chlorotic blood, buffy coat on . xvii 141

palpitations, diagnosis of . xiii 333

Chocolate, constituents of . . xvii 21

Choleraic virus, gradual extension of xxiii 335

Cholera, absence of tears and complaints in . . . . v 169

acidity of saliva and sweat in . xiii 353

analysis of the blood in . vi 447

anatomical characters of . i 236

animalcular theory of . . xxiii 334

appearances on dissection in . iii 110

Aretæus on . . . . . iv 491

Asiatic, Dr. Bellingeri on . v 234

Dr. Mackintosh on . iii 109

Dr. Seymour on . . xxiv 146

effect of civilization on . xviii 253

works on . . . . xxiii 313

at Benares, treatment of . xix 74

-blood, experiments with . v 223

brought into Britain from Riga xxiii 332

charcoal with lard in . . ix 74

chlorine fumigations in . . i 15

coagulated lymph in primæ viæ in . i 238

collapse in, cold affusion in . i 15

condition of individuals favouring xxiii 341

condition of media favouring . xxiii 337

contagious nature of . . v 625

contagiousness of iii 110, ix 74, xiii 353

conveyance of, by persons . xxiii 324

diarrhœa preceding . . xiii 352

difference of English and Indian xiii 352

diseased follicles in . . ii 45

Dr. Bene on the Indian practice in i 15

Dr. Reed's prediction on . xxiii 241

dysentery resembling, in 1670 xxiv 350

effects of saline injections in . iii 112

effervescing draughts in . . iii 182

efficacy of calomel in . . xiii 258

epidemic, affecting animals . iii 256

Dr. Bene on . . i 15

in Holland in 1832-3 . iii 485

in Moscow in 1831-2 . iii 485

fatality of, in advanced age . ix 75

favoured by marching . . xxiii 342

formula for saline injections in iii 112

French commissioners on . xxiii 327

homœopathic treatment of . xxii 580

in animals . . . . iii 110

in Berlin, statistics of . . vi 240

in British troops . . ix 73

Indica, Dr. Williams on . xiii 99

infantile, alumina in . . i 257

infection of attendants by . xxiii 322

infectious nature of . . v 234

in Italy in 1835, report of . ii 279

in London, air heavier before . i 351

in Naples, in 1837 . . v 625

Cholera, inoculation of . . . ix 245

in Paris, graphic account of . i 213

in relation to atmosphere . xxiii 339

in Scotland and America . xxiii 329

intestinal venous system in . i 237

in the Indian army . . xxiii 326

in the United States' army . xvi 43

Italian treatises on . . v 234

isolation and quarantine for . xxiii 330

its connexion with comets, &c. xxi 147

its first appearance in Paris . i 213

its following water-courses . xiii 501

its irregular mode of spreading xxiii 336

lesion of Peyer's glands in . xii 299

Mackintosh on pathology of . iii 111

on remedies for . . iii 111

on saline injections in . iii 112

mortality of, in Venice, 1835-6 v 259

Mr. Parkin on the cure of . iii 181

Mr. Parkin's pathology of . iii 180

nature, and seat of . . v 235-6

of the poison in . . xxiii 316

new species of . . . xvi 558

numerical method in . . xiii 258

occasional exciting cause of . v 234

on the agent causing . . xxiii 333

pathology of . . . xiii 353

probability of life in . . v 292

progress of, in Britain . . x 286

prophylactic rules for . . xxiii 344

report on . . . . vii 149

Russian and Dutch accounts of iii 484

saline injections into veins in . iii 112

singular exemptions from . xxiii 331

state of the air in . . . xxiv 101

blood in . . . xvii 433

statistics of, in Naples, 1836 . v 260

sugar of lead and opium in . v 280

suggestions of Dr. Prout on . xiii 353

symptoms of Asiatic . . v 235

the blood after death in . iii 110

the laws of . . . . vi 576

treatment of . . . ix 74

in India . . . xiii 354

urea in the blood in . . vii 586

use of carbonic acid gas in . iii 179

cold water drinks in . i 15

Indian hemp in . . x 227

Versari on the nature of. . v 169

treatment of . . . v 169

vesicles in the intestines in . i 236

viewed as contagious . xxiii 320, 332

with military movements . xxiii 324

Choleric or bilious temperament . xv 424

Choler, not a fabulous term . . vi 178

Cholestearine in morbid fluids . xi 254

Cholesteatoma of Müller . . xxi 425

Cholosis, characteristics of . . xxii 312

Dr. Horaczek on . . . xxii 312

treatment of . . . xxii 317

Chomel, Professor, eulogy of . ii 33

his clinical lectures on typhoid fevers ii 33

his lectures on medicine . xi 516

CHOMEL, his opinions on fever . iii 390

notice of . . . . . i 212

on rheumatism and gout . vi 352

on typhoid fever, strictures on ii 62

CHOMEL's pathology, Turkish translation of . . . . . iv 397

Chondri decoctum . . . xiii 66

gelatina . . . . . xiii 66

Chondritis, Mr. Wells on . . xiii 171

Chondrus crispus (Carrageen moss) xiii 65

Chorda dorsalis, Dr. Barry on the . xii 570

formation of cells of the . ix 505

nuclei in cells of the . ix 505

Chordee, new explanation of . xii 427

Chorea, blended with mania . . xii 107

and rheumatism, connexion of ix 433

case of, and treatment of . xx 263

cases of, cured by electricity . vi 73

Dr. Babington on . . . xiv 134

his definition of . . xiv 135

Dr. Hall on pathology of . xiv 134

effect of civilization on . . xviii 249

electricity in . . . . . xii 344

fatal, treatment of a case of . xvi 423

frequent among the Jews . iv 505

from pericarditis, case of . ix 432

epidemic, at Cambuslang, 1742 xii 107

convulsive . . . xii 107

instances of partial . . iv 505

its relation to spinal nerves . iv 505

singular modifications of . xii 106

St. Petersburg treatment of . xiv 135

Sti. Viti, treatment of . . xxiv 41

treated by ammon. of copper . xi 235

treatment of . iv 505, xii 107, xiv 135

use of arsenic in . . . ix 269

of splints in . . . xii 284

the cimicifuga in . . x 280

Chorion, formation of the . . ix 15

milk in venous radicles of . iv 458

of the ovum . . . . ix 13

origin of, in mammalia . . vi 514

in man . . . . . xvi 555

in the rabbit . . . xvi 552

villi of the . . . . . xx 302

Choroido-retinitis . . . x 35

Choroid, inflammation of . . xx 282

membrane, two layers of . x 12

of the eye, Van der Kolk on . xviii 49

vessels of, connexions of . xviii 50

Choroiditis, confusion of the term . v 43, 44

the cause of glaucoma . . xiii 505

Van der Kolk on . . . xviii 48

CHOSSAT, M., experiment of, on animal heat . . . xvii 431

on inanition . . . xvii 344, 356

CHOWNE, Dr., his oration . . xiii 219

Christiana, university of . . iv 541

Christians, Eastern, food of . . xvii 41

longevity of . . xvii 41

Christianity of medical men . . xxi 438

CHRISTISON, Dr., his Dispensatory xiii 522

his self-contradiction . . xii 308

CHRISTISON, Dr., mistakes of, on petechiæ xii 310

on disease of kidneys viii 121, x 301, 328

hectic fever . . . xii 95

poisons . . . . xx 317

typhus fever . . xii 293, 308

Chromatic instruments for the eyes xvi 363

Chromatophobia . . . . . xvii 415

Chromic acid, microscopic use of . xi 520

Chromium, experiments with . vii 564

Chrono-thermal system of medicine xv 124

Chronic change, with inflammation ii 17

without inflammation ii 17

changes in the system, symptoms of ii 17

disease, case of, by Schönlein xxi 37

diseases, after injuries, Mr. Travers on ii 11

Hahnemann on . . xxi 232

M. Fourcault on . . xx 102

quasi-epidemic . . xix 27

gastritis, complications of . vii 126

Chronology in the book of Genesis xxiv 481

Churches, American, insalubrity of iv 58

ventilation of . . . xix 3

Church-going in America, evils of . iv 58

Churchyards, stink of, in London . xv 514

CHURCHILL, Dr., on diseases of females vi 86, 97

on diseases of pregnancy . x 544

midwifery . . . . xiv 366

prolapsus of the cord . vii 291

the umbilical cord . . iv 261

turning . . . . viii 597

CHURCHILL, Mr., his Manuals . xxi 479

Chusan, fever and dysentery at . xxii 181

Chyle-cells, origin of . . . xv 271

Chyle, conversion of, into blood . viii 479

corpuscles in . . . xv 271

Chyle-corpuscles, structure of . xiv 262

Chyle, globules of . . . iv 337, viii 479

in birds, generally limpid . iv 342

M. l'Heritier on . . . xvii 427

separation of, from chyme . vi 177

Chylification, culture of . . xvi 223

Drs. Combe and Prout on . ii 378

Chyme, acidity of . . . . . xvi 221

aids to resorption of . . xxiv 277

and chyle, observations on . ii 378

an organic compound . . xvi 221

expulsion of, from stomach . vi 179

separation of chyle from . vi 177

Chymification, a chemical process . vi 177

how effected . . . vi 177

Liebig on . . . . . xiv 512

CIANI, Dr., on gout . . . . ix 238

Cicatrices, from burns, operations on xix 410

in the lungs, new theory of . ix 340

operations for . . . xxi 289

warty, Mr. Hawkins on . . ii 161

Cicatrix, vaccine . . . . . vii 203

Cicatrization, phenomena of . . xxii 24

process of, remarks on . . xxiii 590

CICERO de Senectute, quotation from i 343

Cicindela campestris, *plate of*, dissected i 539

Cider, good Devonshire, described . vi 384

Cilia, in spores of some algæ . . xxiv 224

Ciliæ, in the mussel, movement of . xix 257
Ciliary motions, Dr. Mayer on . iii 465
how investigated . . i 510
in animals, Dr. Sharpey on i 511
nature of . . i 511
Purkinje and Valentin on i 509
in dead animals . . xi 521
in Fallopian tubes . . i 510
in man, Siebold on . iii 502
in oviduct of birds . . i 510
in the brain . . . iii 501
nerves, morbid affections of . xviii 50
processes, diverticula to eye . xxiii 142
Cimicifuga, use of, in chorea . . x 280
Cinchonæ decoctum acidulatum . xiii 85
Cincinnati (U. S.), medical department of college of . . ii 592
Cinenchyma of plants . . . ix 504
Cingalese, Candians, and Vaidahs . xxiv 462
Cinnabar fumigation, new method of ii 555
Circassian beauties, observations on xxiv 465
Circocele, M. Reynaud's operation for viii 558
Circoid aneurism . . . . xxiv 421
Circulation, action of poisons through iii 17
agency of arteries in . . viii 172
capillary, in health . . xvii 568
theory of . . . xvii 568
velocity of . . . xix 262
cerebral, Dr. Burrows on . xxii 408
changes of, in labour . . xix 423
collateral . . . . xxii 104
curious, in vegetable cells . iv 8
Dr. Billing on . . . v 202
Dr. Wardrop's views of . . v 146
in acardiac fœtuses . vii 182, xvii 491
in animals . . . . vii 181
in capillaries . . viii 175, xx 157
arrestment of . . . xiv 599
indirect lateral . . . xxii 105
influence of gravity on . . iv 246
nerves on . . . iii 16
in lungs, renewal of . . xxi 268
in the head, impediments to . iii 324
in vegetables . . . . vii 180
of the blood, in insects . . ii 213
of the lowest animals . . xvii 490
of the spinal cord . . . xiv 379
organs of, in typhoid fever . ii 50
peculiar, in the head . . iii 323
physiology of . . . vii 179
portal, Dr. Willis on . . xiii 262
present knowledge of . . xix 260
re-establishment of, after stagnation xviii 269
remarks on the . . v 337, viii 334
report on the . . . xvii 253
through the lungs . . . ii 518
Valentin on the . . . xi 303
uterine sounds of the . . i 93
Circulatory system in insects . . xx 496
Cirrhose liver, of Laennec, described ii 462
Cirrhosis of liver . . . vii 224
causes of . x 562
Dr. Budd on . xxi 49
Cirrhosis of liver, Dr. Huss on . xxii 474
nature of . . . x 561
other diseases with . . x 562
researches in . . x 560
treatment of . . xxi 51
Cirsocele, cause of, on left side . xii 428
Cities, and towns, mortality in . iii 50
British, hygienic state of . xv 313
cause of higher mortality in . ix 359
in Scotland, typhus fever in . xiv 456
large, evils of, and their cure . xix 127-40
mortality of . . xix 126, 138
Citrate of iron in chlorosis . . xxi 89
Citrene in volatile oils . . . xi 134
Citron wine, formula for . . xvi 220
Civiale, Dr., on the urinary organs xvii 205
his lithontriptic luck . . xii 414
his lithontriptor . . . xii 413
his mode of injecting the bladder vii 167
on calculus . . . xii 388, 394, 412
contraction of urethra . xii 557
diseases of urethra . . xv 346
neuralgia of the bladder . iii 231
urethral cauterization . xv 363
Civilization, effect of, on longevity . ix 359
effects of, on the nerves . . v 540
increase of diseases from . xviii 239
in relation to—ague . . xviii 253
Asiatic cholera . . xviii 253
catalepsy . . . xviii 250
chimney-sweeper's cancer xviii 253
chorea . . . xviii 249
colica pictonum . . xviii 251
congestions . . xviii 251
consumption . . xviii 246
croup . . . . xviii 251
delirium tremens . xviii 250
dysentery . . . xviii 253
fever . . . . xviii 252
hydrophobia . xviii 244, 250
inflammation . . xviii 251
lepra . . . . xviii 254
medicine . . . xviii 255
neuralgia . . . xviii 250
nostalgia . . . xviii 249
ophthalmia . . . xviii 251
paralysis . . . xviii 250
plagues . . . xviii 248
raphania . . . xviii 250
rickets . . . xviii 247
scrofula . . . xviii 247
scurvy . . . xviii 253
smallpox . . . xviii 254
syphilis . . . xviii 248
the dancing mania . xviii 250
the gilder's malady . xviii 250
the plague . . . xviii 252
yellow fever . . xviii 252
its agency on man . . . xv 181
its effects on—longevity . xvii 104
artists and artisans . xviii 243
burning of widows . xviii 244
children . . . xviii 241

Civilization, its effects on—cleanliness xviii 242
contagions . . . xviii 243
cretinism . . . xviii 241
deaf and dumb . . xviii 241
diet . . . . xviii 242
dress . . . xviii 242
duration of life . xviii 237, 246
education . . . xviii 242
endemic agencies . xviii 244
health . . xvii 104, xviii 237
infanticide . . . xviii 241
insanity . . . xviii 239
midwifery . . . xviii 244
military service . . xviii 243
morality . . . xviii 244
mortality . . . xviii 245
nursing of sick . . xviii 243
pauperism . . . xviii 239
philanthropy . . xviii 244
poisoning . . . xviii 242
population . . . xviii 237
prejudices . . . xviii 244
prevention of diseases . xviii 241
prisons . . . xviii 243
punishments . . xviii 243
recovery from diseases . xviii 245
sanitary habits . . xviii 244
sea service . . . xviii 243
the blind, &c. . . xviii 241
the poor . . xviii 242, 245
the supply of food . xviii 242
new diseases from . . xviii 246
Civilized and savage life compared xviii 238, 245
Claessen, Dr., on diseases of the pancreas . . . xviii 388
on the cold-water cure . . xii 189
Clairvoyance, on credulity respecting xix 470
on the evidence for . xix 450, 463
M. Burdin's prize for . . xix 453
pretended cases of . . xix 453
Clanny, Dr., on wourali poison . viii 602
Claridge, Mr., on the cold-water cure . . . xiv 422, 427
Clark, Dr., his evidence on water xviii 495
on purifying water . . xii 522
on diet, &c., commended . ii 387
Clark, Dr. W., his report on physiology i 379
Clark, Mr., his anatomy of the nerves iii 203
Clark, Mr. Le Gros, on the urethra xxiii 414
Clark, Sir James, Dr. Marx on . xv 21, 22
his letters to Tommasini . xv 529
treatise on Consumption . i 70
on Climate . . . . . xii 160-73
consumption, translation of iii 487
detecting arsenic . . xiii 191
medical reform . . xiv 296, xv 529
his Influence of Climate xiv 309, xxii 228
tribute of praise to . . x 243
Clarke, Dr., on putrid dysentery . xxiv 355
Clarke, Mr., remarkable case by . vii 578
Classical learning, advantages of . ix 417
study, exclusive, objections to x 177
Classics, their use to medical men . ix 417
Classification, of diseases, Dr. Lanza's xxiii 61
Dr. Laycock on . . xxi 526
remarks on . . . ix 465
Schönlein's . . . xxi 26
of medical men in Prussia . i 300
Claudicatio, congenital . . xxi 402
Clavicle, case of dislocation of . iii 144
tumour on . . . vii 148
dislocation of, backwards . xii 551
excision of, entire . . . x 568
extirpation of . . vii 147, x 265
luxation of, downwards . . vi 229
sternal end of . . i 584
Clavus hystericus a kind of neuralgia xiii 147
Clay, Dr., on extirpation of ovaria xvi 387
Cleanliness, effect of civilization on xviii 242
importance of, in insanity . xxiv 159
its importance to health . i 331
Cleft palate, case of operation for . ii 498
cause of deafness in . viii 96
knot-maker for . . ii 496
Mr. Bushe on the operation for ii 493
Mr. Fergusson's operation for xxiii 412
new forceps for . . ii 495
instruments for . ii 495-6
observations on . xix 408, 415
on the operation for . xxii 382
origin of . . . xx 134
suture instrument for . ii 496
what prevent operation for ii 493
Cleland, Dr., on tobacco . . xi 512
Clendinning, Dr., on Indian hemp xviii 368
on the heart . . . iii 541, vii 142
Clergyman's sore throat, Dr. Green on xxiv 484
remarks on . . xviii 294
Clergymen, age at death of 1000 . i 200
in the West Indies . . viii 218
Clergy practising surgery, bulls against . . . . . xiv 405
Climacteric disease, observations on xx 272
Climate, and meteorology, treatises on xx 215
advantages from change of . xii 161
agency of, on animals . . xv 181
as a cause of phthisis . . xxiii 437
change of, effect of, on health vi 151
Dr. Armstrong on . . . xviii 221
effects of, on the human race . xxi 377
equable, in British Guiana . i 508
hot, Dr. Dunglison on . . i 346
effect of, on the liver . xii 82
improvement of, by cultivation i 353
indiscriminate choice of . . xii 160
influence of, on health . . vi 148
on phthisis . . . xxiv 107
in relation to diet . . . xvii 44
to malaria . . . xiii 257
to mortality . . . iii 49
to population . . viii 40
its effect on disease . . xiii 423
its influence on scrofula . xxii 147
moral influence of . . . xiv 452
of Brighton . . . . . xvi 500
Cove of Cork, its salubrity . iv 324

Climate of Exeter . . . . vi 383

of United States . . . xvi 34, 35

sanative influence of . . xiv 309

Sir James Clark on . . xii 160

on the influence of . . xxii 228

temperate, effect of, on natives vi 151

varieties of man from . . xv 181

Climates, best winter, English . xii 163

English, account of . . xii 166

in France, account of . . xii 169

in Italy, account of . . xii 170

Cline, Mr., his first case of vaccination vi 481

Clinical communications by Dr. Berndt ii 176

contributions, Baumgaertner's xxiv 137

Dr. Gobee's . . xv 399, 402

Dr. Taylor's . . . xxiv 549

examination, M. Louis on . i 400

inquiry, first step in . . i 400

institution at Griefswald . ii 176

instruction, M. Louis on . i 397, 400

remarks on . . . vii 117

investigation, method of . ii 475

lectures, M. Chomel's . . xi 516

medicine, Dr. Graves on . . xvi 232

Dr. Latham on . . ii 473

of Schönlein . . . xxi 33

observation, principles of . xxiii 186

observations in Berlin . . xxiv 41

pathology, Dr. Lanza on . xxiii 47

report, by Dr. Aldis . . i 490

Dr. Del Chiappa's . . i 249

reports . . . . . xxiii 421

select, by Dr. Barlow . xxiii 419

research, two modes of . . xiii 327

Society, reports of . . xxiii 421, 425

study, method of, by M. Louis i 400

system, in Italian hospitals . xxiv 363

teaching, Dr. Latham on . ii 475, 477

remarks on . . . xx 176

Clinique médicale, by M. Andral . i 188

Clitoris, enlarged, case of . . xxi 85

Cloasma and melema, M. Rayer on . ii 155

Close cavities, pathology of . . xviii 79, 85

serous surfaces of . . xviii 80

Clot Bey, on medicine in Cairo . vi 592

on plague in Egypt i 248, xvi 289, 296

talents and courage of . iv 382

Clothes, conveying contagion far . i 377

sleeping with, on and off . xix 16

Clothing and dress—of soldiers . xxi 173

Dr. Reid's remarks on . . xix 16

harm from excess of . . xxii 466

its relation to hygiène . . i 332

of the young, remarks on . i 332

on military . . . . . xix 384

proper, effect of on health . i 331

winter and summer, remarks on i 335

Clot, the, in arteries after ligature . xxii 94

Cloture, M., on fever in Normandy xi 29

Cloves, oil of, a useful anti-emetic . xiii 69

Club-foot, age for operating on . viii 398

anatomical alterations in . viii 387

causes of . . . . . viii 388

Club-foot, combination of, with hare-lip xiii 5
congenital, causes of . . viii 388-9
table of cases of . . xiii 5
cured by operation . . vi 272
Dieffenbach on . . . xiii 4
his treatment of . . ix 228
different opinions on . . xxi 400
division of muscles in . . viii 397
tendons in . v 285, 288, vi 163
Dr. Krauss on cure of . . ix 227
Dr. Little and others on . viii 385, 405
his nomenclature of . viii 394
his remarks on . . xxi 401
Dr. Mutter on . . . xi 513
frequency of . . . . . xiii 4
history of the operation for . viii 393
knife for operating on . . viii 391
Mr. Whipple's paper on . . viii 405
muscles implicated in . . viii 395
oblique division of tendon in . viii 391
operation for, extension after . viii 392
for cure of . . . iv 539
for, described . . . vi 272
palliative means for . . viii 390
plaster of Paris for . . iii 514
posterior tibial tendon in . xiii 6
section of tendo achillis in . iii 233
state of the bones in . . viii 387
tenotomy applied to . . xxi 182
the defect in . . . . vi 163
treatise on . . . . . xviii 353
varieties or degrees of . . xiii 7
Clutterbuck and Broussais compared v 185
Clutterbuck, Dr., on pyrexia . v 185
Clyster of tartar emetic . . . xv 73
Clysters, Dr. Burne's objections to . x 48
Mr. Mayo on the use of . . v 392
Cnidian sentences, account of . xvii 457
Coagula, bronchial ramifying, *fig. of* xxiii 505
in the heart, description of . ii 325
organization of . . . xx 88
Coagulability, nomenclature for . viii 134
Coagulation, in arteries from ligature xxii 94
inexplicable case of . . xix 252
of the blood, Dr. Polli on . xvii 249
Coagulum in heart before death . vi 547
Coal-gas, cases of asphyxia from . xv 252
of poisoning by . . ix 380
non-explosive, poisonous xv 254, 255
peculiar odour of, cause of . xv 253
poisoning by, symptoms of . xv 254
the blood in . . xv 254, 255
poisonous action of . . xv 254
slow poisoning by . . . xv 255
(Strasburg), composition of . xv 253
when explosive . . . xv 253
Cobbett, William, Thos. Carlyle on xviii 481
Cocculus indicus, British importation of xviii 562
poisonous effects of . . xviii 562
principle of . . . xiv 518
Coccyx, muscles of, Theile on . xiv 377
Cochin leg, account of . . . iii 353
L'Hôpital, account of . . i 281

Cochlea of the ear . . . viii 78, 88

Cock, Mr., his anatomy of head and neck i 541

on catheterism . . . xii 346

congenital deafness . . vii 451

injury of the head . . xvi 312

malformation of the ear . ii 167

paracentesis thoracis . . xviii 343

pneumogastric nerve . . vi 60

Cockburn, Mr., on M'Naughten's case xvi 102

Cocks, Mr., his Operative Surgery v 544

Cocoa, less oily than chocolate . xvii 21

rather astringent . . . xvii 21

-seeds, constituents of . . xvii 21

Coction of humours of Hippocrates . xvii 469

Codeine, discovery of in 1832, by Robiquet ii 207

its qualities and use . . ii 207

Cod-fish, bones of cranial vertebræ, *fig. of* xxiii 484

Cod-liver oil, action of, on human body xiii 198

cautions in use of . . xiii 201

cure of phthisis by . . xix 141

dietetic use of . . xiii 198

diseases cured by . xiii 200, xiv 444

doses of . . . viii 554, xiii 199

Dr. Bennet on . . xiii 197

estimate of its efficacy . xiv 445

experiment with . . xiv 443

four kinds of . . . x 558

history of . . . xiii 198

in rickets of children . xx 564

in scrofula, Mr. Phillips on xxii 153

in tuberculosis . . xiv 443

iodine in . viii 554, x 558, xiii 198

its action as a remedy . xxiii 519

its efficacy in scrofula . xiv 445

medical properties of . xiv 443

modus operandi of . xiii 201

properties of . . . xiii 198

taste and flavour of . xiii 199

the doses of . . . xiv 445

therapeutic effects of . xiii 199

treatment of cats with . xiv 443

treatises on . . . xiv 441

use of in scrofula . . viii 554

use of the three sorts of . xiv 444

Cod-oil, in scrofulous diseases . ix 553

paper on . . . . . x 584

Coffee, acorn, for cachectic children xvii 378

and tea, food for the liver . xiv 519

salubrious effects of . xiv 517

best time for drinking . . ix 242

constituents of . . . xvii 21

-grounds vomit, origin of . i 136

injurious in certain habits . xiv 359

Cogswell, Dr., on iodine . . v 162

Cohesive attraction in chemistry . xi 144

force of food . . . xvii 31

Coimbra, university of . . . vi 284

Coke in the cornea, removal of . xviii 421

Colchicum, tinct. of, for constipation xix 31

action of, in gout . . . xii 73

and lytta, externally . . viii 280

as a preventive of gout . . viii 500

doses of, in fever cases . . iv 250

Colchicum, Dr. Lewins on . . iv 249

letter on . . . iv 565

in gout, hints respecting . xvi 467

its action in gout . . xvi 467

on the kidneys . . viii 500

its effects in fever . . iv 250

its efficacy in gout . . viii 499

its external use in gout . . iv 536

its use in gout . . . xvi 466

rheumatism . . . xx 187

scarlatina . . . v 281

on its operation . . . viii 499

root of, in rheumatic gout . vi 556

trials with, in health . . iv 250

Cold, and heat, in relation to the nerves iii 15

extreme, effects of . . . xi 315

intense, on the effects of . xviii 217

stupor produced by extreme . i 347

use of, in diseases . . . ii 529

in thoracic diseases . ii 531

in typhus fever . . ii 531

Cold and hot applications, Mr. Key on iii 121

liquids in stomach, effects of . xviii 533

lotion, improved mode of applying ii 199

lotions to the head, in fever . xvi 240

Cold affusion, in collapse of cholera i 15

M. Josse's method of . iii 115

remarks on . . . v 359, 360

bath when sweating, safety of xxii 451

dash for furious delirium in fever ii 64

in fever with cerebral inflammation . . . ii 64

in fever, overlooked in Paris ii 64

douche, effects of . . xxii 397

in mania, M. Pinel on . xx 37

foot-baths for sleeplessness . xxii 54

plunge à la Russe . . xvi 471

bath, reviving effect of . xxii 435

water cure, diseases treated by xiv 431

observations on the . xiv 422

regimen in the . xiv 430, 436

treatises on . . . xxii 428

water, fever treated by . . xxiii 269

in fever, fallacy respecting xxii 447

in stomach, effects of . ii 388

its efficacy in wounds . iii 121

its use in surgery . . iii 114

Preissnitz's process with . xiv 429

treatment of wounds by . v 574

Colds and coughs, preservation from xv 541

Coleoptera, curious observation on xxiv 439

Coleridge, his fascinating conversation . . . xxiv 216

Coley, Dr., on diseases of children xxii 548

Coley, Mr., on vesico-vaginal fistula vii 289

Colica pictonum, effect of civilization on . . . xviii 251

fatal case of . . . xx 222

from belts and gloves . xvi 43

from lead . . . xviii 549

M. Chevallier on . . ii 573

use of oil in . . . vii 284

warm water in . . xiv 61

Colic, from copper and lead, diagnosis of x 441
from lead, remarks on . . xvii 416
description of . . x 427
lead, treatment of . . x 445
see Lead Colic.
homœopathic treatment of . xxii 580
of Madrid, nature of the . x 442
Collapse in cholera, cold affusion in i 15
Collectanea Medico-chirurgica . xxii 226
College, medical, in Jamaica . . iii 194
new medical, proposed . . xix 214
of general practitioners . xix 615
of health in Denmark . . ii 298
of Physicians, cannot make doctors xxii 549
new charter of . . xix 552
of Ireland, admissions into i 610
of New York . . . ii 585
of Surgeons in London . . xiv 407
new charter of . . xvii 279
remarks on . . xvii 280
reform in . . . . ix 287
Colleges, medical, multiplication of xiv 411
of the United States. ii 583, 593
Collegiate medical system in London xiii 268
COLLES, Dr., his lectures on surgery xxi 271
on syphilis . . . . . v 1, 27
COLLIE, Mr., and his collection of skulls ix 211
COLLIER, Dr., his London Pharmacopœia . . . . . iv 101, 103
on food in the arctic regions . xiv 508
versus the College of Physicians iv 103-4
COLLINS, Dr., his treatise on midwifery ii 71
on dilatation of os uteri . iv 398
on natural labour . . . . ii 74
remarks on his style . . ii 75
unfair quotations from . . iv 403
unintelligible on one point . ii 81
COLLINS, Dr. Samuel, celebrated in Russia . . . . . i 602
Colloid cancer, anatomy of . . xxi 425
tumours, numerous, case of . xx 100
Collyrium, of iodine . . . xii 258
of vinum belladonnæ . . vii 220
Collyria, Dr. von Ammon on . . x 35
mode of using . . . xi 57
Mr. Jeaffreson on . . xviii 407
observations on . . . xxi 163
saturnine, mischief from . xviii 407
to the conjunctiva, remarks on xxiii 512
Coloboma iridis, appearance of . xvii 235
operation for . . . xxi 308
Coloborrhinocephalus fissilabrus . v 171
COLOMBAT, M., his treatise on women xxi 83
Colonial journals . . . iii 192
medical journals . . . ii 234
Colonization by Great Britain . xiv 454
Colon, contraction of, in dysentery iii 334
induration of, in dysentery . iii 334
limpid urine in, loaded . x 41
mucous coat of, morbid anatomy of xxiv 336
opening the . . xxiii 411, 420
in lumbar region . xviii 464, 471
points in pathology of . . viii 501
Colon, structure of its mucous coat xxiv 335
stricture of, non-malignant . xii 116
Coloration ardoisée . . . i 137
Colossochelys, wonderful remains of xxi 476
Colostrum, and milk, alkaline . vi 184
and mucous state of milk . vi 183
and pus, like quality of . vi 184
case of long continuance of . vi 184
coagulability of . . . xxii 292
composition of . . . xvii 443
of the cow, Dr. Davy on . xxiii 411
progressive change of . . vi 183
the "granular bodies" of . vi 183
Colotomia, operation of . . xxi 329
Coloured glasses for the eyes . xvi 363
Colours, in animal bodies, theory on viii 593
modify absorption of fomites . i 378
morbid sensibility to . . xvii 415
on convulsions, &c., from . xix 302
COLQUHOUN, Mr., on a case of idiocy vi 122
on animal magnetism iii 1, iv 441, xix 428
Coma, causes of . . . . xxii 418
diagnosis of, from coma . . xvii 130
fatal, in jaundice . . . iii 327
from exhaustion, full pulse in . viii 526
remarks on . . viii 526
treatment of . . viii 526
from injuries of nervous system xviii 215
from lead poisoning . . x 456
from pressure on the brain . xxii 405
hysteric, cases of . . xix 437
long persistence of . xvii 501
in anæmic cases . . . xviii 488
in cases of exhaustion . . iii 325
in jaundice, rapidly fatal . i 15
mesmeric, operations during . xxii 477
remark on death from . . xvii 500
sudden death by, in jaundice . xxi 103
Comatose intermittent fever . . viii 62
Comb of the bee, construction of . xi 97
COMBE, Dr. A., his Principles of Physiology . . . i 313, 328
his letter to Mr. G. . . xxi 509
his Physiology . . . xii 513
his Physiology of Digestion ii 368, xiii 219
his suggestion on St. Martin vi 181, 581
on digestion . . . . vi 172
dissent from . . . ii 377
on infancy . x 241-51, 514, xii 228
memoir of . . . . xxiv 581
on Nature in disease . . xxi 505
observing Nature in treating diseases . . . xx 592, xxiii 257
on phrenology . . . xxii 230
on the physiology of health . xvii 530
personal character of . . xxiv 584
works of . . . . . xxiv 582
COMBE, Mr., his Notes on America xv 52
on national education . . xxiii 254
phrenology . . . ix 190, 193
the brains, &c., of nations x 485
the constitution of man . ix 193
the skull of an Indian . x 484

Combustion, case of spontaneous human iv 222

sincipital, use of . . . vii 63

spontaneous . . . xvi 74

case of . . . iii 507

of the body . . . xii 531

remarks on . . . ix 62

Commentaries, Medical, Dr. Paine's xi 383

Commercial men, ill-timed exercise of ii 381

Commissioners for towns, report of xxi 333, xviii 492-3

Commons, House of, lighting of xix 19

ventilation of . . . xix 18

Como, Lake of, goitre at . . viii 113

Companionship to invalids . . xviii 474

Comparative anatomy—aided by geology i 392

cultivated by Aristotle . i 380

Dr. Grant on . . . xiii 217

general fact in . . xxii 497

importance of . . i 537

remarks on . . . i 393

in studying the brain . xxii 489

use of . . . . xvii 423

use of nervous . . xiii 480

utility of . . viii 226, 506

nosography . . . xxiv 82

pathology, Heusinger on . xxiv 81

Comparison, in physical diagnosis . iv 296

Compensation of organs, law of . viii 11

Competition, excessive, cause of . xiv 451

Compilers of truth, merit of . . xi 277

Complexion, diversities of, in mankind xxiv 69

Composition, on exercises in . . x 178

Compositors and pressmen, health of xx 425

phthisis in . . . xviii 499

Compound radicals in inorganic chemistry xi 140

Compounds, ternary and quaternary xviii 519

Compress, the hydropathic, effect of xxii 439

Compression, by depressed bone . xiii 132

cure of ascites by . . . xi 237

from extravasation . . xiii 131

from fracture of inner table of skull xiii 133

in cancer, effect of . . xxi 220

in white swelling . . . xii 512

its use in aneurism . . xx 434

methodical, in burns . . i 580

of brain, convulsions in . . xv 171

from suppuration . . xiii 133

Mr. Guthrie on . . xv 169

Mr. Sharp on . . xiii 130

paralysis in . . . xv 170

stertor in . . . iii 251

trephining in . . . xiii 131

Compressor, arterial, of Segnoroni . xxii 111

microtomic . . . . i 510

Compressors for aneurism . . xx 437

Comradeship, medical literary . xxi 447

Conceptions, sensational, Unzer on xxiv 190

Concretion, stony, in the nose . xi 238

fibrinous, of the heart . . ix 386

gouty, hippuric acid in . . xiv 52

sanguineous, of the heart . ix 549

tophaceous, in rheumatism . vi 371, 373

Concussion, cases analogous to . xviii 216

Concussion, case, miscalled one of . xiii 125

classification of . . . xiii 125

diminution of brain from . xv 164

general, fatal effects of . . xviii 214

general treatment of . . xiii 127

local treatment of . . . xiii 127

Mr. Gama's experiments on . xv 163

Mr. Sharp on . . . xiii 124

objections to Mr. Sharp on . xiii 124

of brain, definition of . . xiii 124

fact relative to . . iii 11

Mr. Guthrie on . . xv 163

on the shock in . . xv 165

remark on . . . iv 144

remarks on . . . v 173

treatment of . . . xv 166

vomiting in . . . xv 166

what must occur in . xv 163

on hasty bleeding in . . xiii 128

opium in . . . xiii 128

Sir B. Brodie on stimulants in xiii 127

state of the pupils in . . xv 167

brain in . . . iv 439

symptoms of . . . xiii 126

breathing in . . . . xv 167

use of mercury in . . . xv 168

purgatives in . . . xiii 128

Condidling, a kind of monomania . x 75

CONDILLAC, philosophy of . . v 83

Condiments, observations on . . xvii 23

use of, unnecessary . . xvii 24

Condition of England question . xix 318

Condylomata, ointment for . . xix 519

structure and site of . . ix 251

three forms of . . . xiii 178

under the tongue . . . ix 251

Confectionery, green, poisoning by xviii 551

Confections, improvement in . . iv 112

Conferva, contagious, in salamander ix 549

Confervæ, in mouth during sleep . xxiii 509

on living frogs . . . xiii 523

Confervoid filaments from the bowels xvii 27

Confinement, its effects on health . i 340

solitary, effect of, on brain . xv 56

Confusos, race of the, in Brazil . x 477

Congenital changes in the body . xxii 337

luxations, treatise on . . xxi 393

Conger eel, respiration of the . xv 209

Congestion, active and passive . iii 60

active, nature and signs of . iii 209

theories of . . . iii 210

a frequent cause of . . viii 182

and inflammation . . . xv 37

and obstruction . . . xxiii 55

cause of, Dr. Williams on the xvii 492

cerebral, active . . . xii 100-1

passive . . . . xii 100-1

differential character of . . viii 195

Dr. Billing, on the cause of . xvii 578

Dr. Canstatt on . . . xiii 333

Hippocrates' explanation of . iii 210

local, treatment of . . . x 65

nature of . . . . . viii 182

Congestion, nature and formation of . iii 209

 observations on . . . . xvii 493-5

 of brain, passive, in children . xxi 383

 of the heart, diagnostics of . vi 134

 on hypertrophy from . . xvii 494

 or hyperæmia, Mr. Mayo on . iii 57

 passive . . . . . iii 211

  effects of . . . iii 211

  readiest seats of . . iii 211

 pathogenesis of . . . iii 210

 Prof. Naumann on . . iii 209

 permanent, effects of . . iii 210

 remedies for, Dr. Williams on xvii 495

 venous, philosophy of . . xi 398

 visceral, common causes of . vii 126

Congestions, effect of civilization on xviii 251

 fibrinous effusions from . . xvii 494

 prevalent in winter . . xxiv 103

Conii cataplasma, substitute for . iv 112

Conjunctiva, anatomy of the . . xi 59

 arteries of the . . . . ii 235

 blueish, in the delicate . . iii 224

 corneæ, inflammation of . xx 278

 cuticular, nature of . . x 36

 diseases of the . . . . xi 60

 discoloration of, by nitrate of

  silver solution . . xi 68

 dividing chemosed . . . vii 156

 excision of the . . . . v 38

 excrescences of the . . xi 65

 extirpation of the . . . x 32

 granular . . . . . viii 232

  Dr. Jungken on . . ii 502, 505

  Mr. Tyrell on . . . xi 62

  treatment of . . . ii 507

 granulations of, crucial incisions in ii 508

 inflammation of the . . xx 273

 inflammations of the . . x 31

 collyria to the . . . xxiii 512

 scarifying the . . . . x 32

 smallpox pustules on . . x 32

 ulceration and mortification of . xx 276

Conjunctivitis, affection of nose in . xxiii 373

 diagnosis of . . . . . ii 345

 gonorrhœal . . . . . x 393

 scarifications in . . . vii 219

 scrofulous, bleeding in . . vii 219

 the blood-vessels in . . vii 218

 treatment of . . . . . vii 219

Connecticut lunatic asylum, report of vii 1, 50

Connexion of certain diseases . . viii 501

Conolly and Seymour, Drs., compared xxiv 156

Conolly, Dr., and Hanwell asylum xi 515

 dedication to . . . . . xii 222

 farewell dinner to . . . viii 304

 his connexion with the Review xxiv 585

  Hanwell report . xi 104, 126, xv 218

  treatment of the insane . xv 25

 on Hanwell asylum . . xi 271

  lunatic asylums . . . xxiv 156

   in Paris . . xix 281

  seclusion of the insane . xi 546

  the Parisian asylums . . xix 594

Conolly, Dr., on treatment of the

  insane . . . . . xiii 274

Conquest, Dr., on tapping the head vi 265

Conscience, difficult cases of . . xxiv 229

 effects of insanity on . . xv 418

 or the moral sense . . . xxiv 227

 the idea of right and wrong . xxiv 228

Conscientiousness, insane, case of . vii 12

Consciousness, cases of alternating . xix 443-5

 direction of to a part . . viii 495

 double, explanation of . . xx 16

 duality of . . . . . xx 5

 seat of . . . . . . xxii 512

Consensual movements, remarks on xix 310

   sources of . . xxii 505

 phenomena, instances of . . xxii 506

Consonance, Dr. Skoda's ideas on . xiv 173

Constantinople, medical school of . iv 389

Constipation, and ileus, cases of . xx 242

 causes of habitual . . . x 45

 croton oil and opium in . . xiii 69

 cutaneous diseases from . . x 45

 effect of bread-pills in . . xxiii 267

 female diseases from . . x 44

 habitual, bougies in . . x 47

  calomel in . . . x 47

  castor oil in . . . x 47

  clysters in . . . x 48

  Dr. Burne on . . . x 40, 48

  effects of . . . v 391

  senna in . . . . x 47

 in females, at puberty . . x 41

 in the great, Voltaire on . . vii 26

 mode of treating . . . vii 478

 obstinate, treatment of . . xiv 61

 the cause of diseases . . x 41

 tincture of colchicum in . . xix 31

 treatment of habitual . . x 45

Constitutional disease . . . xxiv 305

 inflammation . . . . i 552

  Mr. Travers on . . ii 22

 irritation, Mr. Travers on . . ii 1, iii 1

  of two kinds . . . ii 3

Constitution, and habit . . . xxiv 303

 Dr. Lévy on . . . . . xix 56

 hereditary, modified . . v 406

 modifying disease . . . xiii 520

 West Indian, feeble . . ii 15

Constitutions, in relation to accidents iv 187

 morbid, remarks on . . xx 314

Constrictor cunni, anatomy of . xix 516

Consultation, a medical, in Turkey . iv 388

Consultations, medical, in America . iii 278

Consulting practitioners, remarks on x 217

Consumption, absence of, in the navy xv 13

 American statistics of . . iii 526

 causes of . . . . . xx 102

 choice of a climate for . . xii 162

 curability of . . . . xix 140

 cured by ol. asphalti . . xii 432

 deaths from, in England . . xi 425

 Dr. Campbell on . . . xiv 181

 Dr. Furnivall on . . vii 536, xvii 531

Consumption, Dr. John Hastings on . xvi 490

effects of climate on . . xii 160

exciting causes of . . . viii 223

heated rooms in . . . xii 163

hour of death in, tables of . xviii 175

in different classes . . . xx 423

in English women, cause of . xi 425

influence of climate on . . xxiv 107

season on . . xxiv 105

in Malta . . . . . xiii 434

in relation to countries . . xiv 44, 46

to occupations . . xx 426

in the army and navy . . xv 11

West Indies . . viii 219

in workmen indoors . . xviii 499

M. Louis on . . . . . xvi 425

marsh miasm adverse to . . xiv 44

mortality by, in Ireland . . xviii 39

number of deaths from . . xx 428

preservation from . . . xv 541

prevalence of, in the army . viii 225

prevented by diet, &c. . . ii 387

prevention and treatment of i 81-2, xx 108

pulmonary, etiology of . . xiv 225

pure alkalis in . . . xiv 193

remissions in . . . xviii 212

rife among black troops . . vi 150

sea-voyages for . . . xv 14

Sir A. Carlisle on . . . vi 188-9

Sir J. Clark on causes of . i 72

on statistics of . . i 78

treatise on, by . . i 70, 84

statistics of . . . . xviii 499

in Madeira . . . xxiv 107

table of hourly deaths in . xviii 175

treatises on . . . . xv 540

treatment of . . . . . x 549

use of liquor potassæ in . . xiv 196

Consumption, see Phthisis.

Consumptive, residence for, a new . xv 15

Contagion, action of acids on . . xi 447

and infection, distinction of . xv 381

and miasma, Dr. Henle on . ix 398

and yeast, analogy between . xi 445

animate, in syphilis . . xi 219

progress of . . . ix 401

cause of immunity from . . xi 446

chemical action of . . . xi 446

conditions of existence of . xxiii 319

conveyed far by clothes . . i 377

curious cases of . . . ix 571

dormant semina of . . . xxi 359

Dr. Holland on . . . xxiii 313

experiments on . . . . xx 406

from dead bodies, remarks on . i 378

influence of temperature on . i 378

in plague, Clot Bey on . . i 249

instance of sudden . . . v 270

in typhoid fever, M. Chomel on ii 52

its laws and origin . . . i 369

Liebig on the nature of . . xi 445

lurking in localities . . xi 24

material of, organic matter . xi 435

Contagion, matter of . . . xix 363

observations on . . xx 211, 349

organic nature of . . . ix 398

puerperal, Dr. Helm on . . xiii 103

remark of Dr. Shapter on . xii 94

Schönlein's views of . . xxi 21

specific, Dr. Henry on . . i 370

Contagions, distinction between . xxiii 317

effect of civilization on . . xviii 243

materies morbi in . . . xxiii 315

their communication . . i 374

Contagious cells, inoculation by . xx 214

diseases, from parasites . . ix 398-403

M. Magendie on . . viii 315

fever, causes of . . xi 18, 22, 36

generated anew . . xi 4

Stevens on blood in . ii 483

fevers, phenomena of . . xvi 196

matter, Liebig's theory on . xvi 196

Contagiousness of a disease, evidence of xxiii 321

Contagium animatum, idea of a . xv 412

Continent, medical institutions of . i 494

Continuity, congenital solutions of . viii 14

Contracted joints, treatment of . iv 171

Contractile fibrous tissue, atrophy caused by . . . . ii 462

Contractility, muscular, Dr. Reid on xii 277

fibres of . . xiii 164

nervous system in xiii 483

remarks on . xiii 478, 483

of muscle, morbid . . . xvi 418

property of . . . . . v 487

simple, fibres of . . . xiii 164

vegetable, remarks on . . xiii 478

Contraction, gradual extension in . xiii 17

induced, phenomena of . . xxiii 407

of limbs, in apoplexy . . xx 258

in children . . . xxi 471

permanent muscular . . v 140

uterine, means of inducing . iii 525

Contractions, from cicatrices . . iv 171

muscular, in dead bodies . xxiv 279

of joints, from fasciæ . . iv 169

on permanent muscular . . iv 169

operations for . . . xiii 14

Contractura, various causes of . v 140

Contra-stimulant doctrine of Rasori vii 425

Contre-coup, on fracture of cranium by xv 173

Contributors to the Review, remuneration to . . . xxiv 595

valedictory tribute to the . xxiv 597

Contrivances, Divine and human . xvii 449

Controversies of physiologists . iv 78

Controversy, shameful physiological v 622

Contusion of head, money-compress in xi 311

Contusions, their import in legal medicine . . . . ii 415

Convalescence in fever, precepts on xvii 370

Convalescent institution, metropolitan xii 576

retreats in India . . . i 345

Convention, the World's Temperance, proceedings of . . xxiv 515

Conversazione, medical, at Cheltenham iv 560

Conversion, cure of diseases by . xxiii 590

sanative and insanative . . xxiii 590

Convolutions of brain, M. Foville on xviii 432

Convulsion, experiment on . . iii 32

relation of, to spinal cord . iii 32

stridulous, in infants . . xxiii 197

Convulsions, apoplectic, Dr. Hull on xvii 203

endemic . . . . . xii 75

epidemic, Dr. Laycock on . xii 75

epileptic, from exostosis . xvii 203

from colours . . . xix 302

from lead poisoning . . x 457

from spinal disease, case of . xii 123

from the idea of water . . xix 302

in children, Dr. Mauthner on . xxi 391

treatment of . . . xxi 392

in five children . . . viii 595

in 16,434 labours . . . ii 73

interesting case of . . . xii 123

of children . . . . xx 555

of infants, facts on . . . xx 363

case of peculiar . . . xi 234

puerperal, cases of . . . xviii 340

causes of . . . iv 406

delivery in . . . iv 409

Dr. Collins on . . ii 86

Dr. Davis on . . . iii 408

Dr. Meigs on . . . xxi 91

emetic tartar in . . ii 87

ergot of rye in . . iv 409

neglected cases of . . ii 87

on opium in . . . iv 409

Prof. Romberg on . . xvii 422

rare afterwards . . ii 87

rarity of . . . xvii 423

report on . xxiii 289, xvii 539

signs of . . . iv 408

treatment of . . iv 406, xvii 422

Convulsive disease, remarks on . xx 146

diseases, caused by urea in blood? xviii 233

electricity in . . . vi 72

Indian hemp in . . . x 228

spinal source of . . xvi 170

CONWELL, Mr., on diseases of the liver iii 329

COOK, Dr., on consumption . . xv 540

COOKE, Mr., his memoir of Sir. Wm. Blizard ii 205

Cookery, by soldiers, remarks on . xxi 172

changes in food, by . . xvii 36

Cooks, high salaries of . . . xvii 36

Coolings, number of, in hydropathy xxii 444

COOPER, Mr. B., cases of hernia by ix 392

his case of wounded knee-joint ii 198

his Life of Sir A. Cooper xvi 118, 143

his fatal ovarian operation . xx 89

his surgical cases . . . vi 66

on amputation . . . ix 392

air in the veins . . . xx 87

capital operations . . xiv 132

fracture . . . . vii 450

of femur . . . xxiii 425

reparation of bone . . iv 367

stricture . . . . xii 330

of urethra . . xiii 312, 327

COOPER, Mr. B., on the muscles of the eye . . . . . vii 457

operation of, for hernia . . xii 332

COOPER, Mr. S., his Surgical Dictionary vii 526, xi 305-6

COOPER, Mr. W. W., on sight . xxiii 510

COOPER, Sir A., and his professional brethren . . . . xvi 135

and Mr. B. Cooper . . xvi 132

Mr. Hey, of Leeds . . xvi 132

Mr. Lewis . . . xvi 133

William IV . . . xvi 140

anecdote of . . . . viii 535

artists employed by . . xvi 133

as a lecturer . . . . xi 550

as a teacher . . . . x 105

at Paris in 1793 . . . xvi 126

at St. Thomas's Hospital . xvi 125

autopsy of . . . . xii 349

birth and parentage of . . xvi 119

boyhood of . . . . xvi 120

boyish exploit of . . . xvi 121

dinner to, in Edinburgh . xvi 141

Dr. Pirondi on . . . viii 535

early education of . . . xvi 120

examiner at the College of Surgeons xvi 138

false report of his death . . xvi 141

fees given to . . . xvi 135

his ardour and activity . . x 105

attacks of vertigo . xvi 136, 142

attendance on royalty . xvi 137

on the Duke of York . xvi 139

baronetcy . . . xvi 138

boldness and caution . . x 104

character . . . . xi 550

of himself . . . xvi 143

detection of murderers . xvi 134

discoveries . . . xi 551

discussion with Abernethy xvi 128

election to Guy's . . xvi 129

first lectures . . . xvi 125

habits of study . . . xi 551

honours from Edinburgh . xvi 141

from France . . xvi 140

illness and death . . xvi 142

improvements . . . xi 551

influence on surgery . . x 104

marriage . . . . xvi 125

merits as an operator . xvi 143

perforation of the tympanum xvi 131

practice . . . . xi 550

professional improvements x 101

retirement from practice . xvi 139

second marriage . . xvi 140

splendid museum . . x 105

studies in London . . xvi 122

Surgical Essays . . . xvi 137

lectures . . . xvi 126

terror at sea . . . xvi 127

tour in Scotland . . xvi 141

triennial prize . . . xvi 143

tying the aorta . . . xvi 136

the carotid . . . xvi 131

COOPER, Sir A., his work on Dislocations xvi 138
work on Hernia . . . xvi 131
the Breast . . xvi 142
the Thymus Gland . xvi 140
in Edinburgh . . . xvi 123
Life of . . . . . . xvi 118
list of his works . . . xi 552
obituary of . . . . . xi 549
on dislocation . . . ix 391
dislocations and fractures . xiv 28
Edinburgh characters . xvi 123
spermatocele . . . vii 444
the breast . . ix 538, x 101
the testis . . . . xii 515
tying arteries and nerves . iii 154
professor of anatomy . . xvi 127
of comparative anatomy . xvi 136
sketch of his life . . . xi 549
tribute to his merits . . x 101-5
with Mr. Cline . . xvi 121, 125
Copaiba, action of, impaired by fluids i 578
administration of . . . xiii 68
adulteration of . . . xiii 67
and cubebs, mode of action of xi 529
balsam of, new mode of giving ii 243
baneful effects from . . xxiii 508
by enema, in gonorrhea . i 578
capsules of . . . . xiii 68
manufacture of . . xiii 68
eruption from use of . . xiii 67
injected into the bladder . iii 521
lessens activity of pills . . iv 117
Mr. Pereira on . . . xiii 67
new mode of administering . vi 558
poisonous effects of . . ix 268
rheumatism from . . . viii 280
COPEMAN, Mr., his cases of apoplexy xx 197
on apoplexy . . . . xvi 272
Copenhagen, account of . . xiv 112
deaths of workmen at . . xiv 112
degree of M.D. in . . . ii 289
diseases in seasons at . . xiv 120-2
diseases of . . . . . ix 217
workmen at . xiv 114, 115
health of labourers in . . xiv 111
medical faculty in . . . ii 287
institutions in . . ii 285
topography of . . ix 216
scientific establishments in . ii 291
surgical academy in . . ii 293
university of . . . ii 285
COPLAND, Dr, couplet applied to . xvii 504
his Dictionary of Medicine iv 200, xvii 503
on abortion . . . . vi 81
on dysentery . . xxiv 333, 356
on pestilence cholera . . xxiii 313
Copper, ammoniated, in diabetes . ii 180
arsenite of, poisoning by . xviii 551
chemical stains from . . xi 54
colic from, symptoms of . x 441
detection of arsenic by . xvi 275-282
in the system . . xi 53
in milk, sweat, &c. . . xvii 4
Copper, naturally in the body . xi 54, 226
in the system ? . . xviii 549
new process for detecting . xviii 549
preparations of, as emetic . xv 73
poisoning by . . ix 58, xi 53
report on . . . xviii 549
subchloride of, poisoning by . xviii 551
sulphate of, croup treated by . i 568
with tartar emetic . . xv 73
utensils, on electrotyped . xviii 552
Coprolithes as a manure, Liebig on xvii 227
Dr. Buckland's discovery of xvii 227
Coptic origin of the negro race . xxiv 444
Copts, observations on the . . xxiv 443
Copulation, organs of, anatomy of xix 507
CORAY, his edition of Hippocrates xvii 451
CORBYN, Mr., his Indian journal . iii 192
Cord, spinal, pathology of . . ix 437
umbilical, circumvolutions of xix 546
Dr. Kilian on tying the . iii 413
external feeling of . . viii 369
injections into . . iii 244
length of . . . iv 261
pressure on, cause of death from xv 318
reposition of the . . iii 523
sound of pulse in the . i 94
sounds of the . . viii 369
prolapsus of, causes of . xv 315
Dr. Collins on . . ii 90
Dr. Denman on . ii 91
Dr. Michaelis on . ii 90
frequency of . . xv 315
CORFE, Mr., on the kidneys . . x 221
Cork, Cove of, salubrious climate of iv 324
CORMACK, Dr., on air in the veins . vi 455
on fever in Edinburgh . xviii 179, 185
Cornage, or roaring in horses, cause of iii 29
CORNELIANI, Prof., on creosote . i 265
CORNARO, on the diet of . . xvii 41
Corneal conjunctiva, Dr. Römer on
the arteries of the . ii 235
Cornea, acute suppuration of the . xi 73
conical, Mr. Tyrrell on . xi 69
operations for . . xvii 235
coke in, removal of . . xviii 421
diseases of, Mr. Tyrrell on . xi 66
foreign bodies on the, various ii 269
healing of wounds of . . xx 280
inflammation of the . . xx 277
-knife, on Beer's . . . xvii 332
-knives, common, objections to xvii 332
on straight and curved xvii 334-37
layers of the . . . . x 9
Mr. Scott's section of, in cataract xvii 335
nature of inflammation of . xvii 585
nerves of the . . xiv 382, ix 548
opacities of, use of iodine in . viii 523
opacity of, iodine in . . xii 258
treatment of . . . v 35
oval figure of the . . . x 10
purulent infiltration of . x 393
remarks on opacities of the . xvi 271
removing foreign particles from vii 219

Cornea, section of, in cataract . xxiv 367
transplantation of the . . iv 537
ulceration and mortification of xx 279
ulcer of, Mr. Jeaffreson on . xviii 411
ulcers of, Mr. Tyrrell on . xi 67
vessels and nerves of . . xxii 284
Corneitis, hypopium in . . vii 218
Mr. Tyrrell on . . . xi 67
with vesication . . . xi 73
Corn, festered, case of . . . xix 561
Corns, and bunions, treatise on . xix 560
cure for . . . . . x 287
Malgaigne on . . . . x 352
nitrate of silver for . . xix 561
Coronary arteries, on peculiarities of xviii 334
artery, case of rupture of . xi 238
circulation, Dr. Hall on . xiv 198
of the heart . . . xiv 575
on suspension of . . xiv 199
veins, rupture of . . . xx 225
Coroner, anecdote of an ignorant . iv 99
medical information of a . xx 320
Coroner's courts, strictures on . ix 356
inquest, evidence in . . ii 608
inquests in Ireland . . xviii 43-45
medical evidence at ix 52, xviii 45
observations on . . xii 175
remarks on . . . . ii 393
solemn farce in . . xviii 45
specimens of . . xviii 535
on appointment of . . ix 51
their office, duties, &c. . . vii 589
Corpora lutea, before fecundation xvi 526, 557
formation of, report on . xxii 290
nature of . . . xvi 527
observations on . . xiv 574
structure of . . . xiv 584
true and false . . ix 570
virgin, characters of . iv 463
Corpora olivaria in animals . . xxiv 575
Corpora striata, office of the . . xxii 310
Corporations, British, medical, statistics of . . . . . i 306
Irish, medical, statistics of . i 610
Corpses, contagion from, remarks on i 378
fevers from putrid . . . x 213
in the Morgue fastened down . ii 412
medical inspectors of all . xviii 201
moved by evolution of gas . ii 412
Corpuscles, blood, blood-nuclei of . xiv 572
oval of vertebrata xiv 571
smallest, of mammals xiv 570
colourless, of blood . . xv 273
Mr. Addison on . . xx 121
compound inflammation . xxii 20
of the blood . . . xvii 250
Mr. Addison on . . xviii 92
origin of the . . xv 275
red, functions of . . . xv 273
white, adhesive property of . xviii 107
Dr. Williams on . . xviii 106
functions of . . . xviii 569
Corpus callosum, structure of the . xviii 438
Corpus cavernosum penis, anatomy of xix 508
Corpus luteum, Dr. Montgomery on iv 462
M. Baer on the . . i 239
structure of the . . ix 445
Corpus spongiosum urethræ, vessels of xix 509
Corpus striatum, effect of lesions of iv 379
Correspondence with the Editor on
"Young Physic" . . xxii 243
Corrigan, Dr., on aortitis . . v 273
on bruit de soufflet . iii 257, vii 276
opium in rheumatism . ix 274
Corrosive liquids, swallowing . v 439
Corrosive sublimate—albumen as an
antidote of . . . xvi 195
albuminous antidote for . xviii 542
antidote for . . . xviii 553
decomponents of . . viii 361
detection of . . . ix 59
endermic use of . . v 352
galvanic antidote for . xvi 154
poisoning by . . xvi 77, xviii 342
tests for . . . xviii 544
to ulcers . . . ix 553
Corsican animals speckled . . iv 130
Corvisart on diseases of the heart i 425
Coryza, danger of to young infants xv 327
in infants, treatment of . xv 327
Costello, Dr., his Cyclopædia viii 413, ix 539
Coste, M., on embryogeny . . ix 2
Costiveness, causes of habitual . x 45
Dr. Burne on . . x 40, 48
Cottage-grate, remarks on . . xix 16
Cotton, and calomel, in ophthalmia xx 463
in burns, Prof. Velpeau on . i 580
or down of the typha, in burns i 580
treatment of erysipelas by . v 244
Cotton-batting, unhealthfulness of xv 297
-factories, Dr. Ure on . xv 294, 293, 301
growth in . . . xv 295
health of children in . xv 295
Mr. Chadwick on . . xv 306
-mill girls, catamenia in . xv 308
-mills, ample room in . . xv 293
Dr. Taylor on health in . xv 309
health of children in . xviii 147
of Austria, account of . xviii 147
health in . . xv 307
oils in, not unhealthful . xv 298
osseous deformity in . xv 308
phthisis in . . . xv 310
temperature of . . xv 293, 298
work in, not unhealthy *per se* . . . xviii 148, 152
-phthisis, M. Villermé on . xv 298, 312
-pneumonia, M. Villermé on . xv 297
Cotugno, liquid of . . . viii 79
Couching, Mr. Egerton's mode of . xvii 341
Mr. Scott on . . . xvii 341
Sautconnea's mode of . . xvii 342
Coudret, Dr., on animal electricity viii 406
Cough, causing rupture of lung, case of iv 254
from disorder of par vagum . iii 27, 28
irritation of the ears . xvii 414

Cough from tapeworms, case of . xvi 246

in relation to spinal curvature . v 142-3

severe, from elongated uvula . xiv 2

sympathetic, diagnosis of . iv 305

various causes of . . . xvi 246

Coughs, chronic, often gouty in old age xvii 114

Coulson, Mr., on deformities of chest iii 169

on diseases of hip-joint . ii 231, iii 169

of bladder . vi 509, x 247, xv 228

Council of health and education xix 218, 221

in Paris . . v 448

Countenance, beauty of, Sir C. Bell on xviii 445

Counter-action, Dr. Jenner on . vii 61

on the term . . . vii 57

Counter-agents of caustics . . xxii 171

Counter-irritant liniment, Dr. Graves's xx 448

lotion, formula of . . xvi 246

lotions, Dr. Granville's . vii 292

use of ammonia as a . vii 68

Counter-irritants in cerebral affections i 496

Counter-irritation, accidental . . vii 60

by heat . . . . vii 62

cause of relief by . . vii 61

caution as to . . . vii 71

curious case of . . vii 61

Dr. Granville on . . vi 498

in cases of poison . . vii 62

in hydropathy . . xxii 443

natural . . . . vii 58

of scalp, remarks on . vii 64

on the term . . . vii 57

situations for . . . vii 70

treatises on . . . vii 56

Counting, the method of, Louis on . i 403

Country, and towns, mortality in . iii 50

-doctor, a tantalizing . . vi 502

-house, eligible site for . . xix 9

Coup de soleil, pathology of . . xviii 217

Courvoisier, remarks on his case . xvi 84

Coutanceau on pulmonary vapour i 242

Cove of Cork, climate of . . xii 168

salubrious climate of . iv 324

Cowan, Dr. C., his Bedside Manual xiv 536

his medical experience . . i 170

retrospective address . xx 509

translation of Louis on phthisis i 165

work on auscultation, &c. . iii 189

on intermittence . . . iii 162

the brain . . . . ix 222

the stethoscope . . . v 267

Cowan, Dr. R., on fever in Glasgow xi 30

Cowards, desperate fighting of . xii 150

Cow, after-pock in the . . . ix 95

inoculation of, with smallpox ix 80, xiv 51

intestinal invagination in . xii 253

miliary-pock in . . . ix 95

stone or wart-pock in . . ix 95

udder-eruption in . . . ix 95

variolation of . . . x 469

water-pock, or bladder-pock in ix 95

confinement of, in towns, evils of xxi 342

Earl Spencer on gestation of . xi 273

effects of food on their milk . xvii 25

Cow, in Bengal, pustular disease in . vi 486

vaccination, direct from . . vii 198

varieties of pocks in . . vii 190

Cowpock, casual, in man . . x 467

casual, in the cow . . . x 464

in the cow, table of . . ix 89

lymph, primary, procuration of x 467

spontaneous, in cows . . ix 86

spurious white . . . x 467

Cowpocks, varieties of spurious . vii 190

Cowpox, acceleration of . . vii 195

alteration of its quality . . vi 493

and smallpox, affinity of vi 482, 485, 492

coexistent case of . xxiii 307

deviations in . . vi 491

identity of vii 204, 295, ix 76, 79, xiii 560, xiv 51

anomalous, in the cow . . ix 95

at Passy, in France . . ii 251

cause of varieties in . . vi 487

Chronicle, design of . . vi 496

complicated with variola . vii 201

complications with . . vii 200

constitutional symptoms in vii 197-8, 200

dentition in . . . . vii 200

direct from the cow . . vii 198

effect of extraneous fever on . vii 200

experiments on, in France . ii 251

fatal form of, in cattle . . vi 485

from primary lymph . . ix 86

the cow in Denmark . v 209

the grease in horses . i 376

history of . . . . vi 478

in America . . . xxi 362

in Wirtemberg . . vii 186

in cow, description of . . ix 92

effect of age on . . ix 90

effect of season on . . ix 90

from smallpox . . ix 80

influences on . . . ix 88

irregularities in . . ix 94

Prof. Hering on . . ix 76

progress of . . . ix 93

spurious . . . . ix 95

symptoms of . . . ix 91

in cows, from human smallpox xiv 397

influence of temperature on . vii 195

interesting fact relative to . vii 202

-itch . . . . . . vii 201

its identity with smallpox . i 376

Jenner's first difficulties . . vi 479

limit of its protection . . v 209

marks after . . . . vii 203

matter, regeneration of . . ix 76

modified, course and nature of . vii 194

not relied on . . . vii 194

modifications of genuine . . vii 191

Mr. Ceely on . . . . xiv 397

origin of, from smallpox . ix 79

pathological principle in . . vii 200

petechial, case of . . . xv 154

prevalence of . . . vi 485

retardation in progress of . vii 194

Cowpox, secondary eruption in . vii 201

susceptibility for . . . vii 192-3

temporary security by . . vi 491

varicella with . . . vii 201

-vesicle, necessity of watching . vi 492

with chronic eruptions . . vii 202

cutaneous diseases . . vii 202

Coxæ morbus, length of limb in . xxii 78-83

Coxalgia, congenital, luxation by . xxi 397

Cox, Mr., on amputation at hip-joint xxii 111

Crab-shell, unvascular dentine in . xxi 80

Craigie, Dr., his Practice of Physic iii 452

on fever in Edinburgh . iv 247, xviii 179

Craig, Mr., his sneers on other authors viii 468

on protracted labours . . viii 467

Cramer, Dr., on abdominal typhus xii 293

on fistulæ and ulcers . . i 268

Cramming children, bad effects of . ii 383

Cramp of the tongue, case of . . x 557

Crania Americana . . . . x 474

ethnographic collections of . xxiv 381

of animals, remarks on . . xvii 372

Cranial bones, fracture of, in labour xvii 549

slow reparation of . . iii 77

forms, not permanent in races . xxiv 73

nerves of men and animals . viii 377

positions, in labour . . xix 67

saw, a new . . . . . xiv 555

tumours, bloody, of infants . vii 86

Craniology, founded in truth . . xx 34

human, objections to . . xxii 522

of animals, worthless . . xxii 521

of osseous fishes . . . xxiii 483

Craniotabes, age subject to . . xvii 375

convulsions in . . . . xvii 375

Dr. West on . . . . . xvii 566

essential nature of . . . xvii 376

mortality from . . . . xvii 376

of early childhood . . . xvii 371

predisposing cause of . . xvii 377

symptoms of . . . . . xvii 374

tetanic spasms in . . . xvii 375

treatment of . . . . . xvii 378

Cranioscopy, an impossible art . xx 34

Dr. Carus's new system of . xiv 65, 75

Cranio-spinal axis, function of . xxii 510

fluid, observations on . xxi 454

Craniotomy, child's death in relation to xii 488

crotchet in . . . . . xii 487

delay in . . . . . xii 487

extraction in . . . . xii 488

by finger in . . . . xii 487

in contracted pelvis . . xix 417

injecting the skull in . . xii 487

new perforator for . . xvii 539

or embryulcia . . . . xii 486

perforators for . . . . xii 487

survival of a child after . . xxiii 283

use of blunt hook in . . xii 487

Cranium, absence of callus in . iii 77

and face, development of . xviii 446

cancer of . . . . . xxii 310

deficient ossification of . . v 273

Cranium, fracture of, by contre-coup xv 173

flux from ear in . . xv 174

inner table of . . xv 174

the base of . . . xv 173

through the orbit . . xv 178

infant, peculiarities of . . vii 89

loss of part of, case of . . xv 400

morbid growth of in puerperal women . . . . . xix 393

of the ancient Egyptians . xxiv 444

on the blood within the . iii 323-4

quantity of blood within, not fixed xxii 408

structure of, in early life . xvii 371

vascular pressure within . xxii 413

Craquement, an important sign . i 213

Crasis of Hippocrates . . . xvii 469

Creaking sound, in pericarditis . xxiv 550

in chest, when heard i 482

Creatine in broths and soups . xvii 22

Creation, by a general law . . xix 167

picturesque sketches of . . xxiv 281

Vestiges of Natural History of xix 155

Credulity, and scepticism, remarks on xvi 188

of the present day, remarks on xix 473

remarkable instances of . . xix 471

Cremaster muscle, Mr. Curling on the xvii 56

Creosote, caution on its use . . ii 170

different opinions of . . i 265

discovery of . . . . ii 168

diuretic effect of . . . ii 170

Dr. Elliotson on . . . . ii 168

Dr. Root's experience of its use ii 201

external use of . . . . iv 251

has no action on the bowels . ii 170

injection of, in glanders . ii 170

in nervous excitability . ii 169

in pulmonary affections . iii 509

in ulcer of vagina . . vi 235

its power over nausea . . ii 169

its use in asthma . . . ii 169

bronchorrœa . . ii 169

chilblains . . . xiii 69

diabetes . . ii 180

hysteria . . . ii 169

neuralgia . . ii 169

palpitation . . ii 169

pulmonary ulcer . ii 168

method of obtaining . . ii 283

ointment of . . . . vi 270

use of . . . . . iv 120

remarks on the dose of . . iii 509

to arrest vomiting . . ii 169

topical use of . . . vi 270, 235

use of in checking vomiting . xiii 68

Crepitation, absent in typhoid pneumonia i 488

not always in pneumonia . i 487

Creta preparata, best preparation of iv 117

Cretin, derivation of the term . xvii 514

Cretinism, and goïtre, difference of xvii 514

causes of . . . . . xx 213

Dr. Knolz on . . . . xix 372

effect of civilization on . . xvii 241

Guggenbuhl's treatment of xvii 514-15

Cretinism, in India . . . viii 114
not always hereditary . . xvii 516
report on . . . . xx 564
some account of . . . xvii 514
Cretins, of Switzerland, remarks on i 276
Swiss institution for . . xvii 515
various degrees of idiocy of . xxiii 234-5
Creuznach, mineral waters of . xiv 356
CRICHTON, Mr., on lithotomy . iv 539
CRICHTON, Sir ALEXANDER, his commentaries . . xv 459-70
on criminal insanity . . xvi 81, 99
Crime, injustice of punishment for xvi 95
intemperance the great cause of xxiv 517
on motive in commission of xvi 84, 91
Crimes, bordering on insanity . xxiv 240
question of insanity in . . xxiv 241
the usual incentives to . . xxiv 239
Criminal cases, irresponsibility in . xix 36
law of England, looseness of, in xix 35
lunatics in England . . xix 341
investigation, English . . ii 393
French . . . ii 392
responsibility and insanity . xxiv 226
legal test of . . . xv 87, 273
Criminals, bodies of, fighting for . v 453
phrenology of . . . xiii 527
Crises, and critical days, in fever, remarks on . . . ii 41
by pulmonary exhalation . i 246
in fever, on discharges in . xiii 342
in fevers, doctrine of . . x 207
in typhoid fever, M. Chomel on ii 41
pathology of . . . . xvi 216
Crisis, and metastasis, Hufeland on xxiii 593
in disease . . . . xxiv 319
CRISP, Mr., on diseases of blood-vessels . . . . . xxiv 410
Critical age, in females, remarks on xiv 390
of women, in France . xx 138
remarks on . . ii 513
days of Hippocrates . . xvii 469
discharges, delusion of . . xvi 337
terminations of diseases . . xxi 23
Criticism, honest, of this journal . xvi 490
medical, observations on . xxi 447
of some journals . . xvi 490
Croakers on the medical profession xxi 440
Crocodile, dental development in xx 403
Crocodiles and serpents used as food xvii 16
CROMMELINCK, Dr., his anatomy . xiii 519
CRONIN, Dr., his books . . xix 242
Crooked back, cure of a feigned . xviii 122
Cross-breeding, Mr. Walker on . vii 379
CROSS, Dr. J. C., on delirium tremens vi 320, 331
on medical eclecticism . . iv 193
CROSSE, Dr. J. G., his anniversary address iii 208
his case of prolapsed bladder . xxii 319
his inaugural address . . xxi 283
on inversio uteri . . . xx 511
CROSSE, Mr., acari from silex by . xix 170
Crotchet, for craniotomy, form of . xii 487
wire, for abortion, Dr. Dewee's vi 86
Croton oil, affection of brain from use of xi 121
in affections of larynx . ii 554
plaster in Hôtel Dieu . xiv 229
test of purity of . . xiii 70
use of, as a purgative . xiii 69
Croup, adult, remedies in . . v 438
use of alum in . . v 438
albuminous exudation before . xii 109
and tracheitis, difference of . xii 109
and diphtherite, difference of xviii 300
distinction of . . . xv 326
benefit of free vomiting in . xvii 557
bronchotomy in . . . v 438
case of false membrane in . xxiv 47
in the fœtus . . . xvii 549
cauterization in . . . vi 530
cure of . . . . . . xii 246
on chemical principles . vi 446
Dr. Dunglison on . . xviii 301, 302
effect of civilization on . . xviii 251
external use of opium in . iv 243
history of an epidemic . . xv 249
in children, remarks on . . iii 445
-like inspiration of infants . ii 112
M. Billard on . . . xi 205
MM. Barrier and Berton on xv 326
mortality from, remarks on xviii 301
of, in Paris . . . xv 248
M. Valleix on . . . xviii 300
nature and treatment of . iv 243
objections to bleeding in . v 438
observations on . . . xiii 569
on opening the jugular in . v 437
on the false membrane of xi 206, 402
primary and secondary v 437, xviii 301
not contagious . . v 437
relation of to bronchitis . xii 356
report on . . . . xvii 556
secondary, adults subject to . v 437
contagious . . . v 437
spasmodic, as a distinct disease xi 207
remarks on . . . iii 445
thymus gland a cause of . ii 237
tincture of iodine externally in xvii 557
tracheotomy in xii 110, xiii 248, xv 327
failure of . . . xv 251
M. Trousseau on . . xvii 299
successful in . . . xi 526
treated by sulphate of copper . i 568
treatment of . v 437, x 588, xviii 302
by emetics . . . xiii 549
by emetic tartar . . ix 573
use of arm-baths in . . xii 246
emetic tartar in . . v 437
sulphate of copper in . xvii 557
stethoscope in . . v 437
Crowing-children, alarm occasioned by ii 114
-disease, Dr. W. Griffin on . xxi 104
of children, cause of . iii 29
CROWFOOT, Mr., on fracture of spine xvii 98
CROWTHER, Dr., on madhouses vii 2, 48, xi 515
Crude tubercle, description of . xxiii 430
Cruelty to animals, essay on . viii 536

Crumpling, pulmonary sound . ix 313
sound, creaking variety of . ix 314
in pericarditis . . xxiv 551
Cruor of the blood after death . viii 274
Crural hernia, Mr. Teale on xxii 205, 208
ring, anatomy of . . . xxii 206
CRUSE, Dr., on bronchitis of children xii 350
Crustacea, production of lost parts in xx 307
supposed metamorphoses in . ii 214
Crustaceans, on the heart of . . xv 210
CRUVEILHIER, M., on ulcer of stomach vii 241
on sounds of the heart . . xii 533
Cry of children, as a diagnostic . ii 356
Cryptæ minimæ, or Lieberkühn's glands i 526
Cryptogamia, in animals . . xii 557
reproduction in . . . vii 183
Cryptogamic plants in expectoration xxiii 510
Crystalline capsule . . . vi 205
lens, anatomy of . . vi 205, 207
case of dislocation of ii 351
development of . . vi 209
dislocation of . . . ii 350
effect of loss of . . xxiv 368
inflammation of . . xx 282
minute structure of ix 511, 516
polarizing structure in . ii 599
Sir D. Brewster on ii 218, v 602
Crystallization, water of, remarks on xi 145
Cubebs and copaiba, mode of action of xi 529
Dr. Pereira on . . . xiii 70
CULLEN and PINEL on typhus fever xii 296
CULLEN, Dr. Armstrong's sarcasms on i 60
early life of . . . . iv 189
CULLEN, Dr. W., notice of . . i 135
opinions of, on nervous system v 526
CULLERIER, M., his practice in syphilis x 418
Cultivation, effects of, on plants . xvii 25
its relation to malaria . . i 353
CUMMIN, Dr., on infanticide . iv 87
CUMMING, Mr., on the eye . xxiv 330
Cumulus germinativus in blastoderma xvi 547
Cupping, dry, as a remedy . . vii 70
in several diseases . . xiv 219
mode of employing . xii 183
-glass, account of a new . v 252
-glasses, improved form of . xii 184
Cupri sulphas, in asthma thymicum i 258
in softening of stomach . i 258
Cupro-ammoniacal liquor, virtues of viii 364
Cura magna of Bene, in syphilis . i 18
Curative power of Nature, remarks on xxi 252
process, internal, Hufeland on xxiii 592
Cure, Dr. Campbell's meaning of the word . . . . xiv 191
of diseases, effect of civilization on xviii 245
maxims on . . . xxiii 50
on rational principles of . xxi 510
Sir G. Blane on the word . xxii 238
surgical, Mr. Key's idea of . x 341
Cures ascribed to drugs, remarks on xxi 518
Curiosity, a literary . . . ii 301
CURLING, Mr., his cases of varicocele xxiv 328
his treatise on tetanus . . iii 463
CURLING, Mr., on a rare hydatid of liver xi 463
on atrophy of bone . . iv 152
on hypertrophy of fingers . xxiii 413
on the testis, &c. . . xvii 55, 87
on ulcerated duodenum . . xv 155
CURRIE, Dr., his treatment of fever xxii 446-8
his work on cold affusion . xxii 434
Curriers' yards at Montfaucon . xi 14
Curtain lectures, salubrious . . i 357
CURTIS and YEARSLEY, Messrs., conflicts of . . . xiii 510
rivalry of . . . . xiii 509
CURTIS, Mr., Dr. Kramer's strictures on iii 82, 85
his acoustic chair . . . xiii 512
his work on the ear condemned iii 82
on aural surgery . . . xiii 509
on health . . . . . v 380, 403
Curvature, lateral operation for . xiii 566
of the spine, Dr. M. Hall on . xxiii 188
treatises on . . . xii 177
spinal, section of muscles in . xii 283
CUSACK, Dr., his case of pressure in aneurism . . . xix 526
Cutaneous absorption, discussion on viii 313
Dr. Madden on . . vi 332
remarks on . . . v 344
and mucous membrane . . vii 239
apoplexy, case of . . . viii 552
diseases, see Skin Diseases.
arrangement of . . vi 427
classification of . x 549, xxi 492
caustic in . . . v 287
cured by smallpox . xxiii 308
delineations of . . viii 239
Dr. Bene on . . . i 12
Dr. Green on . . . iv 175
from constipation . . x 45
hemlock baths in . . vi 538
illustrations of . . viii 541
ignorance of . . xv 114
local depletion in . vi 428
M. Rayer on . . . ii 147
Mr. Dendy on . . vi 425
Mr. Plumbe on . . iv 175
nature of . . . vi 427
of animals . . . xxiv 85
of infants . . . vii 93
treatises on . . . xv 372
use of heat in . . . xix 184
eruptions, use of sugar in . v 243
exhalation, remarks on . . xx 312
parasite, account of . . xxi 200
perspiration, report on . . xix 567
respiration, importance of . xxii 190
Cuticle, an organized substance . vi 334
forms and arrangements of . xiv 263
intimate structure of . . vi 333
thickening of, Dr. A. Combe on i 329
modes of growth of . . xiv 265
mucus, &c., report on . . xix 257
permeability of, by fluids . vi 334
pigment, &c., report on . . xxi 546
purposes of . . . . xiv 265

Cuticle, structure of . . . xiv 264

vascularity of . . . . vi 333

Cutis pendula, case of hereditary . ii 568

hypertrophy of skin . ii 458

Cutlers, master, advice to . . v 258

M. Chevallier on diseases of . v 257

working, advice to . . . . v 258

Cut throat, case of, by Mr. Liston . v 465

treatment of cases of . v 466

Cutting short disease, remarks on xxi 510, 512

Cuttle-fish, discharge of its ink-bag xi 101

ink-bag, urinary bladder of xi 101

retina of, Mr. Jones on . vi 421

CUVIER and HUNTER, remarks on xv 195, 201

CUVIER on the conditions of existence v 88

Cyanide of potassium, poisoning by xviii 560

use of . . . . . xiii 59

Cyanogen, a compound radical . xi 132

an organic compound . . xi 132

Cyanosis, case of, by M. Baron . iv 207

by Dr. Walshe . . xv 147

fallacious doctrine of . . vi 48

from absence of right lung . vi 220

of forty years' standing . . xxiv 324

of infants . . . . . xi 208

Cycle, hebdomadal or heptal . . xviii 166

Cycles of years, history of . . xviii 173

periodic . . . . . xviii 172

Cyclopædia, American, of Medicine and Surgery . . . i 152

of Anatomy and Physiology . i 536

Natural Science . . . . xii 227

Practical Medicine, notice of i 176

Practical Surgery . . iv 195

Surgery . . . . ix 539, xiii 168

Cyclocephali monsters . . . viii 9

Cynanche parotidea, epidemic of . xxiii 304

tonsillaris, treatment of . . xxiv 138

two kinds of . . . . vii 550

Cynthia mammillaris, fleshy sack of xxiii 494

Cyst, containing spermatozoa . xx 100

hydatid, remarkable, on diaphragm xxiv 180

protruded into labium, case of iii 394

Cysts, connected with the liver . xxii 166

free, in exudation of the pleura xxiv 119

in the pelvis and ureter . . xv 103

serous, iodine injections in . xiii 543

Cystic and common ducts, occlusion of xxi 53

oxide, deposition, Dr. Prout on xi 354

means of recognising . xvi 315

Cysticerci in a boil-like tumour . xii 530

Cysticercus, account of . . . xx 408

case of, in the brain . . xx 84

inoculation with . . . xx 415

Mr. Gulliver on . . . xiv 49

Cystine, chemical pathology of . xx 64

Cystisus laburnum, bark of, poisonous xviii 562

Cystitis, chronic, nitrate of silver in vi 565

instrument for caustic in . vi 566

Cystocele, Dr. Davis on . . iii 396-7

vaginal, new operation for . x 575

Cystometer, pericardial . . . xxii 211

Cysto-plastic, etymology of . . vii 414

Cysto-plastic operation . . . vii 414

Cytoblastema, characters of . . xiii 339

solid and fluid . . . xxii 329

Cytoblasts, description of . . ix 498

functions of . . . xx 164

Dacryocystitis, chronic, Mr. Mackenzie on xi 78
Dacryocystosyringokatakleisis . xxi 310
Dactylius aculeatus in the urethra . ix 442
DAHLERUP, M., on perforation of xiii 516
stomach . . . . . xiii 516
DALMAS, case of murder by . . xix 40
DALRYMPLE, Mr., eulogy on . xxi 283
DALRYMPLE, Mr. J., on coagula . xx 88
on encysted tumours . . xviii 369
organization of lymph . xi 458
spermatozoa . . . xx 84
the placenta . . . xv 148
DALZIEL, Dr., on respiration in sleep vi 584
D'AMADOR, M., exquisite morsel from vi 300
on English authors . . vi 300
pathological anatomy . vi 263
Dames de maison in Paris . . iv 71
Damp, as a cause of disease . . xxii 65
Dampness, phthisis and ague from . xx 105
DANCEL, M., on false anchylosis xviii 353, 363
DANCE on sibilant and sonorous rattles i 482
Dancing, Dr. Kilgour on . . i 357
mania, effect of civilization on xviii 250
DANGER and FLANDIN, on arsenic xiii 183
process of . . . xiii 186
DANIELL, Prof., on chemical philosophy . . . . . xi 129, 150
on physical inquiries . . ix 422
DANIEL OF GADDEN deemed a sorcerer xix 124
DANIELSON, Dr., on lepra in Bergen xviii 346
Danish medical journals . i 227, ii 231
men, amiable trait in . ii 296
small salaries of . ii 295
statistics . . . x 583
pharmacopœia . . . xii 54, 85
D'ARCET, Dr., on multiple abscesses xv 131
Darnel, bearded, in bread, danger from xviii 556
Dartmouth College, New Hampshire (U.S.) ii 587
Dartoid tissue, M. Burggraeve on . xxiii 143
Dartos, various opinions on the . xvii 56
Dartre rongeante, on cauterizing . xv 36
treatment of . . . xv 36
Dates, recent analysis of . . xvii 20
used as food in the East . xvii 20
DAUBENY, Dr., on education . x 542
on the atomic theory . . xi 129
DAVIDSON, Dr., his Thackery prize
essay . . (App. to vol.) xi 1
his treatise on diet . . xvii 1, 54
his Glasgow report . . v 607
DAVIDSON, Mr., on lacerated perineum viii 284
DAVIES, Dr., on diseases of children xxiii 237
DAVIES, Mr., on pathology and surgery viii 512
on the use of iodine . . viii 512
DAVIS, Dr., faulty style of . . xi 151
his error on urethral orifice . iii 394

Davis, Dr., his system of midwifery iii 392, xii 461

objectionable advice of . . iii 408

on acute hydrocephalus . . xi 151

on mercury in amenorrhea iii 399, 400

opinion of his work on midwifery iii 411

Davy, Dr., his anatomical researches xii 129

his experiments on blood . viii 270

works of Sir H. Davy . viii 545

on meconium . . . . xx 93

organic diseases . . vii 575

quarantine . . . xvi 289

the basilar artery . . vii 574

colostrum of the cow . xxiii 411

blood . . . . . viii 279

after death . . viii 272

temperature of fishes . ii 217

vesiculæ seminales, &c. vi 551

Davy, Sir H., bodily decline of . viii 545

his safety-lamp, observations on xv 82

ill, from mental exertion . i 343

on anæsthesia from nitrous oxide xxiii 550

the works of . . . . viii 545

Dawlish, as a residence for invalids xiv 477

Day, Dr., his translation of Simon xx 206

his Vogel's anatomy . . xxiii 250

Dead bodies, diseases from putrid . x 213

effects of soil on . . xv 519

infection from, remarks on i 378

mode of preserving . ix 397

moved by evolution of gas ii 412

putrid, on vapours from . xxiv 249

time for putrefaction of . xv 519

Dead, disgusting violations of, in London . . . . . x 215

on medical respect for the . xxi 442

shocking neglect of, in Ireland xxiv 285

Deaf and dumb, Dr. Fowler on . xvii 242

effect of civilization on . xviii 241

in Brunswick . . . iii 255

jurisprudence of . . x 151

man's friend . . . viii 69, 71

neglect of, in Austria . iii 255

person, history of a . . iii 227

dissection of a . . iii 227

physical defects in the . iii 256

remarks on teaching the iii 255

teaching, to speak . . viii 102

Deaf dumbness, Dr. Kramer on . iii 100

Deafness, abstinence and inunction in iii 85

affection of auditory nerve in . viii 101

a frequent cause of . . xxiv 23

and dumbness from quinine . x 586

anemic, removed by stooping . xvii 140

on arnica flowers for . . iii 85

bleeding for . . . iii 85

blisters for . . . iii 84

caused by vitiated air . . xviii 501

congenital, pathology of . vii 452

degrees of, in ear-diseases . xxiv 31

douches into the ear in . . iii 84

Dr. Kitto on . . . . xxi 188

drops and injections in . . iii 84

electricity for . . . iii 84

Deafness, emetics in . . . iii 85

galvanism for . . . iii 84

in cleft palate . . . viii 96

intractable nature of . . xiv 62

issues and setons in . . iii 84

Messrs. Yearsley and Curtis on xiii 509

Mr. Cock on the cause of . ii 167

Mr. Swan's observations on . iii 83, 100

on mineral magnetism for . iii 84

moxa and actual cautery for iii 84

nervous, acetous ether in . iii 99

case of, cured . . iii 99

on diagnosis of . . iii 98-9

Kramer's remedy for . iii 99

remarks on . . . viii 101

treatment of . . iii 98-9, xxiv 39

two forms of . . . iii 98

purgatives for . . . iii 85

Russian vapour-baths for . iii 84

salivation for . . . iii 85

table of lesions causing . . viii 99

on tartar emetic ointment in . iii 84

Turnbull's annihilation of . xv 494

use of oxygen gas in . . xix 233

various specifics for . . iii 84

warm and medicated baths for iii 85

Death, after wounds, not from . vi 540

age at, of different classes . xx 422

announcing of, to patients . xv 423

apparent, Mr. Taylor on . ii 425

beginning with the blood . xvii 501

by hanging, on the cause of . ii 418

Prof. Devergie on . . ii 418

by many small injuries . . iii 251

by plug in the throat, signs of ii 414

causes of sudden, Dr. Alison on xviii 214

in England . . xviii 200, 206

dark hue of sclerotica, as a sign of iv 198

diagnostic signs of . . iv 198

Dr. Hull on . . . . xvii 204

from blows, medico-legal case of iii 248

peas in the rectum . . xiv 576

reduced temperature xvii 354, 502

influence of periods of day on . xxiv 116

in Ireland, on the causes of . xviii 35

little suffering commonly in . viii 274

mean age at, in 42 professions i 198

of Quakers at . . . xviii 505

modes of, classification of . xvii 500

molecular and systemic . . vii 174

natural and unnatural, Henle on xxiv 320

nature of . . . . vii 174

on the phenomena of . . xv 428

pictures of past life at . . xii 150

process, analysis of the . . xx 345

punishment by, remarks on . xxiv 237

signs of . . . . . iv 197

before putrefaction . . iv 198

somatic or systemic . . vii 174

sudden, causes of . . . x 424

in jaundice . . . xxi 103

modes of . . . xviii 214

vigour of mind at . . . xv 428

Deaths, and births, diurnal periods of xxiv 114

Deaths, by violence, in England . xv 79, 81

curious causes of violent . ix 357

in England, and Wales, in 1837 ix 352

reports on . . . . ix 344

of a family, from peculiar disease xv 151

registration of . . . . xviii 205

sudden, on inquests on . . xv 82

in England, remarks on . ix 356

statistics of, in Milan x 420

suspicious, English practice in ii 393

French practice in . . ii 392

violent, in England . . ix 357

in 1840 . . xxi 3

Debility, a cause of inflammation . i 8

irritative, oxide of zinc in . vii 567

true and false, nervous . . xiv 337

Debout, Dr., his midwifery plates . xiv 538

De Candolle, M., his botany . iv 1, 3

on organs of plants . . viii 243

Decayed meat, disease from, at Zurich xv 238

Decay, prevention of, by salts . xi 442

Deception and chicane, on medical xxi 451

Dechambre, M., on structure of the lungs i 233

Decidua, cuplike elevations on the . ii 600

early development of the . viii 42

little sacs on . . . iv 458

formation of the . . . ix 24

in extra-uterine gestation . x 587

M. Baer on the . . . . i 239

structure of the . . . xx 303

Decillion, figured and illustrated . xxi 230

Deckmann, Dr., Biographical sketch of vi 290

obituary of . . . . . vi 290

De Claubry and Montault, MM., on typhus . . . viii 428, 430

Decoction of ballota lanata . . i 567

Decomposition, on chemical . . xi 143

on spontaneous . . . xix 251

Decrepitude and old age, Dr. Smith on i 323

Dedication, ingenious mode of . x 475

Dee, Dr. Arthur, history of, in Russia i 600

Deer's tears, medical effects of . xi 233

Defecation-tube, mode of using . vi 560

Deformed pelvis, natural delivery in i 607

Deformity, its prevalence in factories xv 308

from burns, Dr. Mütter on . xviii 525

rheumatic, myotomy in . . xiii 566

Degenerations, Dr. Lanza on . xxiii 57

Deglutition, the action of . . v 508

excitor nerve of . . . viii 265

M. Longet on . . . xiii 227

nerves concerned in . . v 508

of a coin . . . . . vii 255

œsophageal nerves in . . xvii 178

spasmodic difficulty of . . iii 72

De Graaf's vesicles of the ovary . ix 7

Degrees, in Dublin, number of, in 10 years i 610

medical, in Belgium . . iv 282

in Holland . . . vi 280

scheme proposed for . x 203

Delaroque, M., on typhoid fever xii 293, 297

Deleau, Dr., on diseases of the ear viii 69, 71, 95

Delegated existences, Dr. Prout on . xxi 120

Deleuze, M., on animal magnetism xix 428

Deligation of arteries, Lisfranc on . xiv 21

Delirium, acute, effect of bleeding in xx 19

in the insane . . . xxi 418

pathology of . . . xx 18

and mania, diagnosis of . . x 132

chronic, lunar influence on . xx 22

cold affusion in . . . xiv 228

Dr. Hull on . . . . xvii 203

from lead . . . . . x 455

furious, cold dash for . . ii 64

in fever, treatment of . ix 473, xvii 370

in meningitis, cases of . . xiv 228

typhoid, cause of . . . vi 144

Delirium-tremens, a definition of . vi 321

and gastritis . . . xii 103

bleeding in . . . vi 328

cases of . . . . xx 262

chronicum . . . . vi 327

civil disability in . . x 136

classification of . . xxiii 606

common in Russia . . iv 278

critical sleep in . . vi 324, 327

digitalis in . . v 242, vii 265

dose of opium in . . v 242

Dr. Blake on . . . x 550

Dr. Munk on . . . v 600

Dr. S. B. Peason on . vi 320

Dr. Stokes on . . ix 476

Dr. Ware on . . . xxiii 603

Dr. Watson on . . xvii 128

effect of civilization on . xviii 250

emetic tartar in i 421, vi 330, xv 403

emetics in . . . vi 330

etiology of . . . vi 322

fatal terminations in . xxiii 605

febrile nature of . . vi 327

following epilepsy . . vi 325

from coffee . . . vi 322

narcotics . . vi 322

opium . . . vi 322

red lavender . . vi 322

spirits . . . vi 322

strong ale . . vi 322

wine . . . vi 322

wounds, &c. . . vi 323

in America . . . ix 69

Berlin . . . xiv 561

British troops . . ix 69

Copenhagen . . ix 218

different places . . xiv 118

relation to age . . vi 323

relation to season . vi 324

relation to sex . . vi 324

the Indian army . . xxiii 119

West Indies . . ix 69

medicines in . . . vi 329

nature of . . . xx 31

opium in . . vi 331, xvii 129

paroxysm of . . . xxiii 604

pathology of . . . xvi 220

precursory signs of . . vi 324

procuring sleep in . . xxiii 605

Delirium-tremens, remarks on the term . vi 321
remote causes of . . vi 323
slow respiration in . . vii 264
statistics of . . . vi 323
symptoms of . vi 325-7, xxiii 603
synonymes of . . vi 320
the four stages of . . vi 325-7
thesis on . . . xvi 506
to be left to nature . . xxiii 606
treatment of i 421, vi 328, vii 268, viii 595, ix 477
tremor in . . . vi 327
two cases of . . . vi 322
two kinds of . . . ix 476
use of acids in . . vi 329
calomel in . . vi 330
cold in . . vi 328
varieties of . . . vi 321
works on . . . vi 320
Delivery, by the feet, Dr. Bush on . v 579
case of, after episioraphy . iii 524
of unconscious . . v 255
curious mode of promoting . vi 593
in privies, remarks on . . vii 138
on inducing premature . . ix 36
signs of . . . . . iv 465
report on . . . xx 529
spontaneous, mechanism of . xiv 368
streaks in breasts after . . iv 465
uterine vessels after . . vi 90
Delphinia, Dr. Turnbull on . . ii 499
Delusion in insanity, Dr. Prichard on xvi 82
jurisprudence of . . . x 141
Demagnetization, observations on . xix 479
Dementia, acute, morbid anatomy of xx 23
aspect of countenance in . vii 10
causes of, tables of . . vii 9
irresponsibility in . . . x 148
or incoherence . . . vii 9
propensity to theft in . . x 148
simple common . . . xx 24
sudden, curious case of . . x 168
from emotion . . . x 168
validity of deeds in . . . x 147
Dendy, Mr., his philosophy of mystery xii 519
on diseases of the skin . . vi 425
Denis, M., his crude hypotheses . vi 446
on the chemistry of the blood . vi 431
Denman, Dr., defended . . iv 405
Denman's aphorisms, by Dr. Ryan ii 523
Denmark, account of hospitals in . iii 565
medical profession in . . ii 295
popular statistics of . . ix 214
present state of medicine in . ii 285
Royal Board of Health in . ii 298
Medical Society of . . ii 297
small medical fees in . . ii 295
state of medicine in . . iii 565
Dens sapientiæ, disease of jaw from viii 423
Dental decay, cause of . . . xvi 223
Denticles in fœtal whales . . xxi 68
Dentinal pulp, calcification of . xx 387
cells of . . . . . xx 386
Dentinal pulp, development of . xx 384
Dentine, formation of . . . xx 384
minute structure of . . xiv 270
of fishes, structure of . . xx 395
origin and formation of . . xx 378
unvascular, in crab-shell . . xxi 80
vascular and unvascular . . xx 379
Dentists, qualification of, in Prussia i 608
Dentition, analogy of, to puberty . xii 115
a process of development . xii 115
connexion of diarrhœa with . xx 359
with cowpox . . . vii 200
Deontology, medical, Dr. Simon on xxi 438
Deota, an Indian village, account of viii 111
Depilatory ointment of M. Petel . xiii 244
powder of M. Petel . . xiii 244
secret, of M. M'Mahon . ii 153
Depletion, local, with tonic treatment xvii 483
Deposits, urinary, Dr. G. Bird on . xx 58
Derangement, mental, Dr. Moreau on xxiii 217
Derivation and revulsion, remarks on ii 173
Dr. Gondret on . . . vii 56, 73
remarks on the term . . vii 57
Derma, erosive ulcer of the . . iii 556
Dermalgia, rheumatic . . . xiii 234
Dermatolysis, case of hereditary . ii 568
hypertrophy of skin . . ii 458
Dermis, anatomy of the . ii 431, vi 333
congestive inflammation of . xv 380
depositive inflammation of . xv 391
dubious opinions on the . ii 431
effusive inflammation of . xv 384
inflammation of, from animalcules xv 397
mode of minutely dissecting . ii 432
or cutis, description of the . xv 379
papillary layer of the . . xv 379
squamous inflammation of . xv 394
suppurative inflammation of . xv 388
Dermo-skeleton, observations on the xxiii 473
Dervish, manipulations of an Asiatic iv 393
Descemet, membrane of, inflammation of xx 279
Deschamps, M. F., on the astragalus xviii 124
Descriptive anatomy, study of . x 190
Desgenettes, biographical sketch of vi 290
Desiderata in the medical art . xxii 563
Design, argument from, objectors to xxi 111
arts of, use of, anatomy in . xviii 442
in nature, inference from . xxi 110
the arts of, on the study of . xviii 451
D'Espine, Dr., his annual report . xxi 274
on the weight of prisoners . xxi 278
Desruelles, M., on syphilis . v 1, 25
Desire and aversion, Unzer on . xxiv 191
Desires and aversions, action and nature of . . . . . xxiv 198
and passions, remarks on . xxiv 238
Dessenius, his character of Paracelsus xiv 149
Destitution in relation to fever . xviii 512
Destruction of the soft parts, general ii 399
Destructive maniac, anecdote of a . xxiii 236
processes, remarks on the . xxiii 380
Determination, cause of, explained v 202
Development, asymmetrical . xiii 557

Development, by assimilative process . i 389

Dr. Clark on the theory of . i 389

in general, report on , . xix 588

law of eccentric . . . viii 8

M. Serres' theory of . . viii 10

of mammalia, plates of . . xviii 233

of the ovum . . . . . ix 1

organic, laws of . . . vii 176

process of . . . . . v 82

progressive . . . . vii 178

doctrine of . . . xix 174

remark on the term . . v 338

St. Hilaire's ideas on . . i 391

theory of arrest of . . viii 10

centrifugal . . . viii 8

centripetal . . . viii 8

Developments, taking, reprobation of xiv 71

Deventer, college of . . . vi 280

DEVERGIE, M., account of . . ii 390

on carbonic acid . . . xii 264

on detection of arsenic . . xv 251

on forensic medicine . . ii 390

on the milk of nurses . . xviii 155

question in court to . . ii 408

DEVILLE, his collection of heads . ix 210

Devonshire, cider, good, described . vi 384

salubrity of . . . . . xx 523

Devon, South, affections alleviated by xiv 476, 477

as a residence for the consumptive . . . xiv 477

climate of . xiv 470, 473, 477

fever in . . . . . xiv 474

flora of . . . . . xiv 471

frost, snow, and hail in . xiv 473

geology of . . . xiv 471

healthiest season in . xiv 474

hygrometrical constitution of xiv 472

physiognomy of . . xiv 470

quantity of rain in . . xiv 473

relative frequency of diseases in . . . . . xiv 473

sickliest season in . . xiv 474

temperature of . . xiv 471

various diseases in . . xiv 475-6

warmth and moisture of xiv 471

winds in . . . xiv 473

DEWEES, Dr., on abortion . . vi 81

Dextrine, a product of starch . . xi 144

DHUNWANTARI, the Indian physician . . . . xxiii 521, 530

Diabetes, ammoniated copper in . ii 180

analysis of blood in . . ii 281

urine in . . ii 282

and renal disease, compared . ii 227

case of, cured . . . i 467

concomitants of . . . xi 340

confusion from the word . xi 340

cured by diuretics . . . iv 254

Dr. H. Bell on . . . xiv 538

Dr. Stanley on . . . xiii 259

effects of diet on . . . xxii 224

malaria on . . . xi 339

emetics good palliatives in . ii 180

formation of sugar in xi 338, xiv 510, xvii 442

Diabetes, medicines in . . . xi 341

non-salivation of food in . xi 341

proper diet in . . . xi 340

sugar found in the blood in ii 281, vi 446

treatment of . xi 340, xii 334, xiv 539

use of ammonia in . . xii 334

creosote in . . . ii 180

tinct. mur. ferr. in . xi 265

Diabetes insipidus, different forms of vii 159, 160

mellitus, case of, cured? . xiv 247

or melituria . . . vii 164

state of kidneys in . xxiv 45

treatment of . . . xx 270

use of magnesia in . . vii 165

saccharine, researches on . xiii 539

Diabetic blood, analysis of . . vii 457

sugar in . xvii 432, xviii 369

Diachylon in burns, Prof. Velpeau on i 580

Diagnosis, and causes, of diseases . vii 119

bedside manual of . . xiv 536

composite and comparative . xx 316

difficult case for physical . iv 297

difficulties of . . . vi 108

Dr. Hall on, notice of . . i 204

Dr. Watson on . . . xvii 124

Dr. Williams on . . . xvii 500

empirical, remarks on . . xx 309

fanciful, from uterine discharges ii 515

improvements in . . . vi 302

medical, the art of . . xx 308-16

M. Piorry on . . . vi 128

Mr. Tyrrell on errors in . iii 149

of chest diseases, books on . iii 184

physical, on comparison in . iv 296

the eye in relation to . . xix 367

uncertainty of . . . iv 478

Diagnosist, qualifications of a good xx 310

Diagnostic investigations, Remak's xxiii 503

Diaphoresis, critical, remarks on . viii 491

curative effects of . . xxii 450

Diaphoretics, observations on . xvi 194

two positions respecting . viii 492

Diaphragm, action of, in labour . xix 62

in respiration . . xvii 256

adhesions of the . . . xxiv 180

anomalous motions of . . xxiv 168

atony of, causes of . . xxiv 176

case of laceration of . . iv 260

changes in position of . . xxiv 169

clonic spasm of . . . xxiv 174

convulsive action of . . xxiv 173

disease of, symptoms of . . xxiv 168

diseases of . . . . xxiv 166

divisions of, position of . . xix 105

ectopia of viscera through openings in . . . . xxiv 179

height of the . . . iii 542

hernia of the . . . xxiv 178

importance of the . . . xxiv 167

inflammation of . . . xxiv 178

Magendie's experiment on . v 134

malformations of . . . xxiv 178

ossification of, case of . . xiv 562

paralysis of the . . v 424, xxiv 176

Diaphragm, parasitic formations on the . . . . . . xxiv 180

rheumatism of the . . xxiv 177

rupture of the . vii 455, xxiv 172

case of . . . vii 455

spasmodic action of . . xxiv 173

tonic spasm or tetanus of . xxiv 175

tremulous motion of . . xxiv 173

wounds of, with cases . . xxiv 170

Diaphragmatic hernia, fatal case of iv 479

Diarrhœa, and cholera, of infants . xx 559

and vomiting, cerebral . . ix 475

bilious, observations on . xvi 236

cerebral, observations on . xvi 236

chronic, morbid anatomy of . xv 95

cured by oranges . . . xix 31

Dr. Baillie's prescription for . iii 445

essay on . . . . xxiii 304

from fins of smoked salmon . xvii 25

from malarious atmosphere . xviii 510

homœopathic treatment of . xxii 579

in America, treatment of . xix 30

in children, stupor after . i 27

in typhoid fever, its origin . ii 47

morbid anatomy of . . xv 95

deposits in . . . xiv 545

nux vomica in . . . i 256-7

of infants, deep yellow . . iii 444

observations on . . xx 359

treatment of . . iii 444, xxiv 47

particular form of . . . ix 481

peculiar, of stokers . . xiii 231

Diastase, animal or salivary . . xxi 563

a secretion in vegetables . . xi 144

Diathesis, error of Dr. Campbell on xiv 192

influence of, on organs . . vi 306

M. D'Amador on . . . vi 305-6

Rasori's application of the term i 418

Diatheses, transition of, into others xi 357

various, M. Bonnet on . . xxii 62

Diceras, the, a rare intestinal worm xiii 532

Dick, Dr., on derangements of digestion . . xi 223

on diet and regimen . viii 243

on porrigo . . . vi 425, 428

Dickson, Dr., and Shakspeare . xv 125

basis of his doctrine . . iii 470

condemnation of his book . iii 472

exposition of his doctrine . viii 532

his electrical doctrine . . xv 129

emulating Midas . . xv 131

"fallacies" . . . xv 124

fallacy of physic . . iii 469

multitudinous cases . . xv 129

opinion of the profession . xv 124

resignation to his fate . xv 124

magnificent award to . . xv 130

on unity of disease . . viii 531

specimens of his style, &c. . iii 471

Dictionaries, medical, French . . i 178

medical, German . . . i 179

Italian . . . . i 180

Spanish . . . . i 180

Dictionary, biographical . . xix 530

new . . . xiv 536

Dr. Copland's Medical . iv 200, xvii 503

Edinburgh Medical . . i 178

Medical, by Dr. James . . i 176

by Dr. Motherby . . i 177

Dr. Palmer . . xx 207

Dr. Parr . . . i 177

of Medical Terms, Hoblyn's i 517, xix 245

Dr. Palmer's ii 508

Dicynodon, canine tusks in . . xx 403

Dieffenbach, cruel experiment of v 169

cures of squinting by . . ix 558

his cure of wry-neck . . vii 560

mode of treating club-foot . iii 514

operation for new nose . vii 398, 400

operations on the face . vii 250

Operative Surgery xxi 285, 333, xxiii 1

surgery . . . . . xi 194

on breaking a knee straight . xiii 15

cure of old fractures . . xiii 239

division of tendons . . xiii 1, 28

old dislocation . . . ix 555

plastic surgery . . . vii 386

prolapsus uteri . . . iii 512

ruptured perinæum . . vi 536

stammering . . . xii 209, 257

strabismus . . . x 570-2

vesico-vaginal fistula . . iii 511

orthopedic surgery of . . xi 195

physiological error of . . xiii 25

some deficiencies of . . xiii 7

too partial to knife . . xi 196

Dierbach, Dr., his materia medica viii 353, 362

Dietaries for children, remarks on . xvii 48

paupers, remarks on . xvii 48

prisons, remarks on . xvii 49

observations on . . . xvii 47

Diet, absurd practices in regard to . ii 369, 371

and digestion, daily errors in . ii 368, 371

and food, Dr. Pereira on . xvii 1, 54

and regimen, Dr. Dick on . viii 243

Dr. Schultz's rules of . xvi 217

popular works on . xviii 115

attention to, in bilious complaints vii 479

bread-and-water, effects of . iv 192

disproportionate, illness from . viii 528

Dr. Paris on . . . . v 380, 398

Dr. Robertson on . . . v 380, 397

effect of civilization on . . xviii 242

effects of evil habits of . . xvii 53

enormous, in the country . ii 379

errors in, Mr. Macilwain on . ii 182

importance of, in nervous cases viii 528

impoverished, evil effects of . ii 385

in infancy and childhood . xxiii 299

in London life, Dr. Seymour on xxiv 143

in lunatic asylums . . . xxii 358

in military prisons . . . xxiii 168

insufficient, indigestion from . xvii 348

loathing of food from . xvii 348

in the British navy . . xvii 48

in the Russian hospitals . . iv 274

Diet, in the tropics, Mr. Jeffreys on xvii 153

irregular, evil effects of . . ii 371

its bearing on morals and intellect xvii 52

law respecting . . . v 401

low, bad effects of protracted . v 389

meagre and generous . . viii 524

necessity of bulk in . . xvii 51

of nurses, remarks on . . ii 221, 383

soldiers . . . . . xix 385

the indolent, errors in . ii 372

the labourer, its effects on him ii 372, 384

the rich and poor, remarks on xxiii 577

prevention of disease by . . xi 446

propriety of varying . . v 401

rules of . . . . . xvii 39

at watering-places . . xiv 358

system of, by hydropathists xxii 437, 442

the practitioner's dependence on xvii 51

three kinds of, insufficient . viii 527

universal systems of, irrational ii 383

vegetable, recommendation of . xviii 115

treatise in favour of . xxi 148

Dieterich, Dr., on mercurial disease v 2, 29

Dietetic principles, importance of . ii 369

Dietetical regimen in indigestion . xvii 51

Dietetics, functional or organic . xx 131

three considerations in . . xvi 224

treatises on . . . . . xvii 1, 54

Dietz, his edition of Hippocrates . xvii 451

Digestibility of food . . . . xvii 31

Digestion, acid a product only of . xvi 221

action of saliva in . . . xviii 529

and diet, daily errors in . . ii 368

artificial, Dr. Todd on . . iv 281

experiments on . . iv 201

galvanism in . . . vi 527

saliva necessary to . . xvi 221

chemical nature of . . xxiii 378

chemistry of . . . . xix 268

derangements of . . . xi 223

diseases of organs of . . xxiii 304

disordered, diseases from . xiv 330

disorders of . . . . xiv 330-1

Dr. Blondlot on . . . xviii 529

Dr. Carpenter on . . . xiii 485

Dr. Combe's physiology of . xiii 219

Dr. Paine's philosophy of . xi 397

Dr. Philips's galvanic theory of ii 136

Dr. Prout on . . . xxi 109

Eberle's physiology of . . iv 201

experiments on . . vi 172, 527-8

favored by good teeth . . ii 373

function of . . . . xxiii 392

general ignorance on . . ii 368

Hunter's views on . . . vii 524

ideas of the ancients on . . xv 446

influence of bile on . . xxi 563

of eighth nerves on . . viii 478

of nerves on . . . xix 473

Liebig on the process of . . xiv 307

Mr. Paget's report on . . xxi 557

Mr. Mayo on . . . v 380, 388

new experiments on . . xvi 221

Digestion, observations on . . xiii 493

of kinds of food, time of . . xiii 493

of plants, Dr. S. Smith on . v 382

only a chemical process . . xv 143

of the stomach . . . vii 524

physiology of, by Dr. Combe . ii 368

products of . . . . xvii 435

report on . . . . xvii 257

rules for promoting . . xvi 223

saliva the chief agent in . . xvi 222

second, by the cæcum . . v 402

in the cæcum . . xvi 217, 224

secondary, Dr. Prout on . . xi 333

the mind in relation to . . xv 417

Valentin on . . . . . xi 303

Digestive derangement, diagnosis of vii 119

organs, chemistry of . . iii 541

deaths from disease of . ix 355

diseases of . . . xiv 331

inflammatory products in . xxiv 124

J. Hunter on . . . xv 205

of animals, diseases of . xxiv 82

proper culture of . . xvi 217

principle, best test for . . iv 204

modus operandi of . . iv 203

nature of . . . iv 204

observations on . . xxi 561

process, Dr. Prout on . . xvi 481

researches on . . . xxi 561

system, motions of . . . xi 295

Digitalis, account of, by Dr. Johnstone i 193

causes of its variable action . iii 510

cumulative effect of . . xi 509

endermic use of . . . v 352

formula of, for epilepsy . xi 509

important remarks on . . xxiii 417

in anasarca, formula of . . iii 432

in diseases of the heart . . xx 255

infusion of, remark on . . iv 114

its effect on the heart . . i 264

on the uterus . . . ix 560

its use in delirium tremens . v 242

in fever . . . . v 189

M. Lombard on the action of . iii 510

purpurea, case of poisoning by xviii 564

tolerance of . . . . . xi 509

use of, in epilepsy . . . xi 509

in insanity . . . xxii 57

Dilatation, artificial, of os uteri . iii 413, iv 260, 400

of lymphatics, case of . . ii 459

of os uteri, Burns wrong on . ii 84

of right auricle, diagnosis of . vi 134

sudden, of strictures . . v 572

Dilatator conchæ, muscle . . xiv 376

nasi posterior, muscle . . xiv 376

Diluents, on the misapplication of . xvi 197

on the uses of . . . viii 504

Dimorphous primary bodies . . xi 142

Dimsdale, Baron, and Catherine II i 605

Dingo of Australia, cranium of the . xxiv 64

Dinner, a medical, at Cheltenham . iv 558

at Edinburgh, anecdote of . xix 11

Dinner-pill, Mr. Mayo's . . v 390
of Dr. Burn . . . x 47
Dioptric instruments, Dr. Pereira on xvi 362
Dioptron of Paulus Ægineta . . xiv 209
Diploma, medical, in Paris, examinations for . . . i 495
Diplomas, Irish surgeons', number of in ten years . . . i 610
surgical, at Edinburgh, 1835-6 iii 292
at London, 1835-6 . . iii 292
Dipso-mania, confirmed drunkenness x 136
Diphtherite, and croup, difference of xviii 300
distinction of . . . xv 326
of Bretonneau . . . v 437
Diphtheritis, cauterization in . xxiii 376
diagnosis of . . . . xvii 556
epidemic, account of . . xxiii 304
report on . . . . xvii 556
treatment of . . . . v 438
Directory, London and Provincial Medical . . . . xxiii 612
the London Medical . . xx 208
Disarticulations, M. Lisfranc on . xv 43
Discharges, chronic, mineral waters in xiv 340
in uterine disease, on diagnosis from ii 515
Dischidia rafflesiana, the pitchers of iv 14
Discoloration, black, a morbid state i 136
liquiform . . . . i 136
stratiform . . . . i 136
Discoverers, the rival . . . v 621
Discoveries, scientific, in Germany . iii 582
Discovery, on priority of . . ix 96, 105
Discretion in medical men, remarks on xxi 448
Discutients to tumours, remarks on xiv 4
Diseased action, Mr. Davies on . viii 518
actions, secondary, from injuries, &c. ii 9
Disease, analysis of the phenomena of xx 346
and death, cost of, in Lancashire xxi 355
and health, Dr. Richter on . xv 411
at Zurich, from decayed meat. xv 238
complicated, singular case of . i 250
course of . . xxiii 52, xxiv 315
and seat of a . . . xxiii 42, 52
curious endemic, in Turkey . iv 393
definition of . . . . xxiv 297
determinate course of . . xxi 510
disposition to . . . xxiv 301
Dr. Gully's theory of . . xv 408
Dr. Laycock on the natural history of . . . . xxi 525
Dr. Williams, on the nature of xvii 473
duration of, in relation to danger i 408
eclectic treatment of . . xv 412
elementary forms of . . i 117
evolution of, Dr. Marx on . xvi 474
form of, Professor Lanza on . xxiii 54
Gendrin's definition of . . x 56
general and physical signs of . viii 467
German theory of . . . xiv 328
heat, as a cause of . . . xiii 348
hereditability of . . . xxiv 303
idea and nature of . . . xxiv 297
Liebig's theory of . . . xiv 520
Disease, modification of, report on . xxi 545
moral causes of . . . vi 201
new theory of . . . xv 540
of cæcum, French writers on . i 503
often lurks long in the system ii 6
on doing nothing in . xxiii 258-60
on the mind mastering . . i 191
organic doctrine of . . vi 296
origin and progress of . . x 56
periodicity in . . . xxiv 317
predisposing causes of . . xx 340
primary elements of . . xvii 475
process of . . . . . xxiv 298
proximate elements of xvii 474, 475, 486
recovery from . . . xxiv 318
rhythmical and recurrent . xxiv 317
seat of, Prof. Lanza on . . xxiii 53
secondary elements of xvii 474, 475, 486
simple treatment of . . xv 405
subjectivity of . . . xiii 260
susceptibility to, Dr. Marx on xvi 475
symmetry of, remarks on . xxiii 136
symptomatic and idiopathic . xxiv 305
symptoms of . . . xxiv 304
the best physiological teacher xxiii 171
the flaw in old theories of . vi 299
transition of into another . xxiv 319
Diseases, acute, and chronic . . xxiv 315
five stages of . . . xxiv 317
inflammatory . . . xxiii 61
cachectic, in animals . . xxiv 87
chronic, causes of . . . xx 102
quasi-epidemic . . xix 27
classification of, Schönlein's . xxi 26
combination of . . . xxiv 345
cure of, by conversion . . xxiii 590
by Nature . . . xxii 562
danger and duration of . iii 563
distribution of, into classes . xxiii 61
Dr. Laycock on classification of xxi 526
Dr. Lanza's classification of . xxiii 61
effect of civilization on . xviii 237, 239
feigned and factitious . . xviii 117
from morbid animal food . xvii 25
Gendrin's classification of . x 57
general registration of . . iv 280
in England, relative prevalence of ix 352
inflammatory . . . xxiv 110
in relation to temperature . xxiv 107
local and general . . . xxiv 305
modes of treating . . xxi 92
mixed treatment of . . iii 223
natural history and treatment of . . . . . xxiii 257, 585
natural order of . . . xiii 202
nature of, unity of idea on the xiv 330
numerical method of studying i 168
occurring but once in life . viii 502
of children . . . . xxiii 237
of Landsend and Worcester . vi 8
of man and animals, compared xxiv 82
of organic and animal life . x 56
of the head, in India . . xxiii 119

Diseases of the heart . i 424, xxiii 169

of the stomach . . . xxiv 273

viscera, Dr. Chapman on . xix 21

uterine appendages . . xxiii 295

women . . . . . xxiii 239

on the connexion of certain . viii 501

organic, relative prevalence of vii 575

prescribing for names of . ii 474

preventing, effect of civilization in xviii 241

structural, elements of . . xvii 498

transmissible from animals . xiii 28

treatment of chronic . . x 76

typical and non-typical . xxiv 316

Dislocated humerus, reduction of . vi 163

thigh-bone, case of . . iv 143

Dislocation, and fracture, of ankle . xiv 41

congenital, of femur . . xii 259

of humerus . . . x 566

dissection of a humeral . . xii 455

of carpus forwards . . x 51

remarks on . . . vi 309

of cervical vertebra . . xiv 31

clavicle backwards . xii 551, xiv 41

clavicle, Velpeau on . . i 584

femur on pubes, case of . viii 286

forearm, in children . . v 284

head of the fibula . . xiv 40

hip-joint, spontaneous . xiv 59

humerus, reduction of . vi 231

singular . . . xv 402

neck, case of . . . vi 543

radius forwards . . xiv 42

shoulder-joint, seat of . i 585

shoulder, myotomy in . xiii 21

old, fatal reduction of . xiv 41

the lens, case of . . xiv 252

tarsus . . . . vi 538

tenotomy in . . xiii 21

thumb, case of . . . xiv 231

ulna . . . . . xx 138

old, incision of tendons in . ix 555

periods for reduction of . xiv 33

with fracture, reduction of . xiv 32

Dislocations, and fractures, Sir A.

Cooper on . . x 103, xiv 28

axillary . . . . xii 455

congenital, frequency of . xxi 393

of femur . . . viii 559

fatal complications of . . xiv 31

in general . . . . xiv 30

in infants, experiments on . xxi 395

of femur, unreduced . . xiii 420

of hip-joint . . . . xiv 34-9

in children . . . xiv 31

of shoulder, incomplete . . xii 454

of the astragalus . . . xviii 124

mobility in recent . . . xiv 30

old, division of muscles for . xxi 332

section of muscles in . xi 195

section of tendons in . . ix 230

spontaneous, cure of . . iii 129

of femur . . . xiv 554

of the hip . . . xvi 486

Dislocations, subscapular . . xii 456

statistical researches on . . xiii 245

treatment after . . . i 583

undue interference in . . xi 222

Disorganization, process of . . xvi 209

Dispensaries, charitable, in India . xix 72

in Ireland, account of . . ii 186

state of . . . . xiii 513

Dispensary case, mortifying issue of xxi 248

medical officers, Irish . . xiii 514

Dispensatory, Dr. Christison's . xiii 522

Disposition to disease . . . xxiv 301

Dissecting aneurism, Mr. Crisp on . xxiv 418

rooms, Parisian, health in . xi 16

Dissection, in Paris, history of . v 452, 454

remarks on anatomical . . viii 240

salubrity of . . . . v 454-5

wounds in, treatment of . . xi 310

Dissection-wounds, disease from . xiii 95

treatment of . . iv 140

use of iodine in . viii 523

Dissections, anatomical, ancient . xxiii 95

Dissolvent and irritant remedies . xxiii 49

Distoma hepaticum, observations on xx 416

Distortion of spine, Mr. Ayre on . ii 603

Distress, a cause of fever . . xi 24

District medical officers, plan of . xv 343

Diuresis, with excess of urea . . xi 343

Diuretic mixture for dropsy . . xxi 370

powers of ballota lanata . . i 567

Diuretics, Dr. Seymour on . . iii 433

endermic use of . . . v 350

for producing absorption . xiv 10

observations on . . . xvi 193

rules for exhibition of . . xx 69

scale of utility of . . . iii 433

with excitants . . . xvi 201

Divers, time of, under water . . xxii 165

Diverticula, intestinal, remarks on . iv 169

M. Burggraeve on . . . xxiii 142

of the intestinal canal . . xiv 360

Divine attributes, Mr. Burnett on . vii 227

contrivances, perfection of . xvii 449

Dixon, Mr., on anæsthesia . . xxiii 413

on tumour on fifth nerve . xxiv 327

Dmitri Minaja, a lithotomist in Russia i 604

Docimastic test, a new . . . xvi 149

Dockyard-labourers, health of . xiv 111

-workmen at Copenhagen . xiv 11

Dockyards, mortality in different . xiv 114

Doctorate, medical, in Prussia, requisites for i 296

Doctor of medicine, title of . . xxii 549

Doctors, native, in India, jealousy of xix 77

of the old and new school . x 300

*v.* the assurance companies . xiii 578

Dodd, Mr., on varicose veins . . ix 575

Doepp, Dr., on diseases of children xxi 467

Dog, eye of, morbid anatomy of . xvi 384

mad, no fear of water in . . xiii 51

varieties of . . . . . xxiv 63

Dogs, domestic, hiding their food . xix 309

run wild, loss of power of barking in xxiv 64

wild, observations on . . xxiv 65

Dog's flesh, use of, as food . . xvii 16
Doherty, Mr., case of his suicide . vi 473
Dohm, Dr., on fumigations in pertussis i 574
Dolichocephalæ, or long heads . xviii 373
Dolomite in relation to goître . viii 113
Dolor cerebralis . . . . . xvii 418
Domestic animals, diseases of . xxiv 82
origin of races of . . xxiv 58
Donellan, Dr., on erysipelas xiii 373, 382
Donné, Dr., on milk of nurses vi 181, xviii 155
on pulse, respiration, &c. . ii 246
on urine . . . . . xii 539
Donovan, Mr., on quinina . . xii 569
on vinum ferri . . . . xii 568
Dordogne, epidemic sweating sickness at xviii 162
Dorsal muscles, division of . . xii 253
Doses, homœopathic infinitesimal . xxi 228
of medicines, observations on . xvi 203
Dothinentery, Andral on rhonchi in iv 304
auscultation in . . . . iv 304
engorgement in . . . . iv 304
Dotted cells and ducts in plants . ix 503
Douche, cold, effects of . . . xxii 397
use of, in insanity . . . xi 125
Douches into the ear, in deafness . iii 84
Dover, his frightful doses of his powder iv 118
his powder, infant poisoned by xviii 557
Dowler, Dr., his experimental researches . . . . . xxiv 279
Downie, Sir A. M., on mineral waters . . . . xiv 309, 320
on the spas of Homburg xiv 310, 320
Draco volans, account of the . vi 101
Dracunculus in India, account of . xii 429
Dragoons, 14th Light, in India, Mr. Moffat on . . . . . xxi 375
Dragoon Guards, mortality in the . viii 224
Drainage, and sewerage, of towns xxi 333, 348
in towns, expense of . . xviii 494
of towns, report on . . xviii 494
works of, history of . . xxi 336
Drake and Wood, Drs., their journal iii 195
Drake, Dr., biographical notice of ii 305
Drake, Dr. Nathan, his death . ii 305
Draper, Dr., on organization of plants xx 153
Drawings of morbid anatomy . vii 224
Dreaming, and insanity, identity of xxiv 191
observations on . . . xxiii 223
suggested, remarks on . . xix 481
Dreams, psychical phenomenon in . xix 467
Drenching system, remark on . xxi 440
Dresden Lying-in Hospital, statistics of ii 273
Dress, and clothing, of soldiers . xxi 173
effect of civilization on . . xviii 242
flannel or linen, in percussion iv 293
of children, remarks on . . ii 126
of the fair Americans . . v 412
Dressing, after operations, Sir C. Bell on vi 156
education of idiots in . . xxiv 14
sores, on Lisfranc's mode of . xv 46
wounds, French mode of . xiv 15
M. Josse on . . . iii 126
routine practice of . . v 458
Dressings, impermeable . . xx 462
Drinking, cerebral affection from . iii 305, 310
much at meals, effect of . . ii 388
spirits, evil effects of . . i 364
water before and after meals . v 401
Drink, quantity of, requisite . . xvii 42
stimulating, on abstinence from ii 388
Drinks, acidulous . . . . . xvii 21
alcoholic, &c. . . . xvii 22
aromatic or astringent . . xvii 21
Dr. A. Combe on . . . ii 387
gelatinous . . . . . xvii 21
intoxicating . . . . . xvii 22
or liquid aliments . . . xvii 21
slops, or ptisans . . . xvii 21
Dropsical effusions, chemical qualities of . . . xvi 151
urea in . . . . vi 249
Dropsy, after scarlatina . . . xvii 221
remarks on . . v 363
without albuminuria xi 240
after scarlet fever, Dr. Stark on iii 262
albuminous urine in, disquisition on ii 224
articular, emetic tartar in . xi 247
ballota lanata, a cure for, in Siberia i 567
case of walking off a . . xxi 371
caused by suppressed perspiration ii 226
causes of . . . . . iii 60
condensed pathology of . . iii 60
condition of the blood in . xvii 150
Dr. Bene on . . . . i 20
Dr. Chapman on . . . . xxi 369
Dr. Osborne on . . . . ii 222
Dr. Seymour on . . . . iii 429
encysted, Magendie on . . viii 318
new method of curing . viii 318
of ovary . . . vii 465
from anemia of the kidneys . iii 548
from deficient albumen . . xvii 152
in cardiac disease, treatment of vi 390
in children, Dr. Wolff on . xiv 202
inefficacy of cantharides in . ii 549
in relation to intemperance . vi 18
juice of elder-root in . . vi 221
liability of innkeepers to . vi 19
of pericardium, position in . xii 281
pineal gland, case of . . vi 268
scarlatina, the kidneys in . v 364
the ducts of the glands . v 181
the ureters . . . v 182
on a peculiar kind, in Ireland iii 548
opinions of Dr. Christison on . x 323
on the nature of . . iii 429
ovarian, bruit de soufflet in . vi 97
danger of tapping . . xxiv 513
Dr. Ashwell on . . iv 373
extraction of sac in . vi 97
Mr. Lee on . . . xxiv 512
on tapping in . . . iii 139
treated by compression . xxiii 296
pathology of . . . . . viii 319
relative prevalence of . . vi 17
renal, on the cause of . . xii 119

